# Genome Mining-Guided Discovery of Two New Depsides from *Talaromyces* sp. HDN1820200

**DOI:** 10.3390/md23030130

**Published:** 2025-03-18

**Authors:** Xiao Zhang, Luyang Liu, Jiani Huang, Xingtao Ren, Guojian Zhang, Qian Che, Dehai Li, Tianjiao Zhu

**Affiliations:** 1 Key Laboratory of Marine Drugs Ministry of Education, Ocean University of China, Qingdao 266003, China; zhangxiao_ouc@163.com (X.Z.); liuluyang0223@163.com (L.L.); hjn7700@163.com (J.H.); xingtao_r@163.com (X.R.); zhangguojian@ouc.edu.cn (G.Z.); cheqian064@ouc.edu.cn (Q.C.); 2 Laboratory for Marine Drugs and Bioproducts, Qingdao Marine Science and Technology Center, Qingdao 266237, China; 3 Sanya Oceanographic Institute, Ocean University of China, Sanya 572025, China

**Keywords:** depsides, *Talaromyces* sp., heterodimer, anti-inflammatory effects

## Abstract

Depsides and their derivatives are a class of polyketides predominantly found in fungal extracts. Herein, a silent nonreducing polyketide synthase (TalsA)-containing gene cluster, which was identified from the Antarctic sponge-derived fungus *Talaromyces* sp. HDN1820200, was successfully activated through heterologous expression in *Aspergillus nidulans*. This activation led to the production of two novel depsides, talaronic acid A (**1**) and B (**2**), alongside three known compounds (**3**–**5**). The further co-expression of TalsA with the decarboxylase (TalsF) demonstrated that it could convert **2** into its decarboxylated derivative **1**. The structural elucidation of these compounds was achieved using comprehensive 1D and 2D-NMR spectroscopy, which was complemented by HR-MS analysis. Talaronic acids A and B were firstly reported heterodimers of 3-methylorsellinic acid (3-MOA) and 5-methylorsellinic acid (5-MOA). All isolated compounds (**1**–**5**) were tested for their anti-inflammatory potential. Notably, compounds **1** and **2** exhibited anti-inflammatory activity comparable to that of the positive control. These results further enrich the structural class of depside natural products.

## 1. Introduction

Depsides were first mentioned by Emil Fischer and Karl Freudenberg in 1910 to describe a molecule consisting of two or more phenolic acid derivatives connected by an ester bond [1]. The depsides are dominantly distributed in lichen, fungi and plants. Due to their remarkable bioactivities such as anticancer, antibacterial and antiviral, the depsides have attracted chemists’ attention for over a century [2,3].

**Figure 1:**
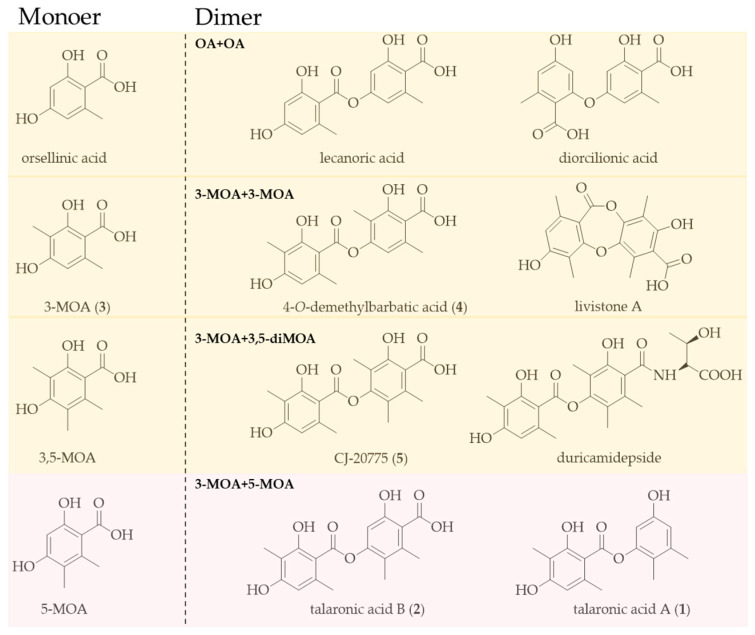
Orsellinic acid, 3-MOA, 3,5-MOA, and 5-MOA heterodimers.

Depside compounds are primarily biosynthesized through the dimerization of orsellinic acid (OA) and its derivatives [4]. Representative structural examples are lecanoric acid [5] and 4-*O*-demethylbarbatic acid [6] (Figure 1), which are formed through the homodimeric coupling of OA and 3-MOA, respectively. However, to our best knowledge, only one kind of heterodimer CJ-20557, which was isolated from *Aspergillus duricaulis* CBS 481.65, is composed of one molecule of 3-MOA and one of 3,5-dimethylorsellinic acid (3,5-diMOA) connected by an ester bond. Due to the structural diversity of OA and its derivatives, it is likely that more heterodimers have yet to be discovered.

**Figure 2:**
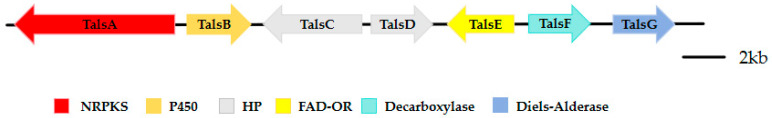
The Tals gene cluster from *Talaromyces* sp. HDN1820200.

**Figure 3:**
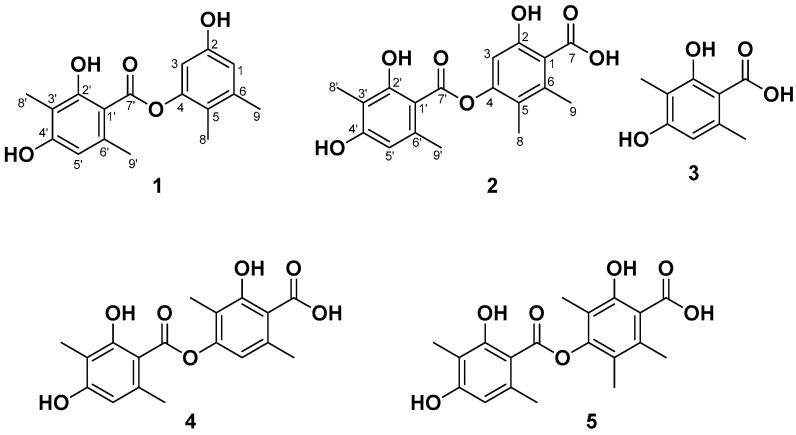
Chemical structures of isolated compounds **1**–**5**.

During our ongoing genome mining work on the fungal strain *Talaromyces* sp. HDN1820200, we discovered a seven-genes cassette (named Tals BGC as shown in Figure 2), which encodes a nonreducing polyketide synthase (TalsA), a P450 monooxygenase (TalsB), two hypothetical proteins (TalsC and TalsD), a flavin-dependent monooxygenase (TalsE), a decarboxylase (TalsF) and a Diels–Alderase like enzyme (TalsG). The TalsA shows moderate similarity with Pre6 [7], which is lecanoric acid synthase. Further phylogenetic analysis of TalsA indicates that it may be responsible for the biosynthesis of an unknown depside. To investigate their functions, all seven genes (TalsA-G) were transferred into *Aspergillus nidulans* A1145 and two novel dipsides, talaronic acid A (**1**) and B (**2**), along with three known compounds (**3**–**5**) (Figure 3) were isolated from the transformant *AN-TalsA-G*. The talaronic acid B (**2**) was identified as a new heterodimer depside, which was composed of 3-MOA and 5-MOA units.

## 2. Results

### 2.1. Bioinformatic Analysis of the TalsA and Tals BGC in Talaromyces sp. HDN1820200

**Figure 4:**
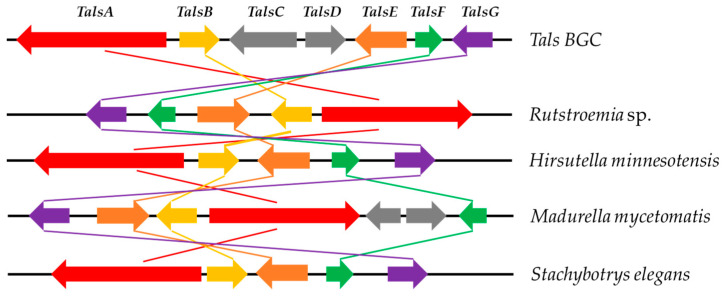
Comparison of the Tals BGC to homologous BGCs in other fungus.

**Figure 5:**
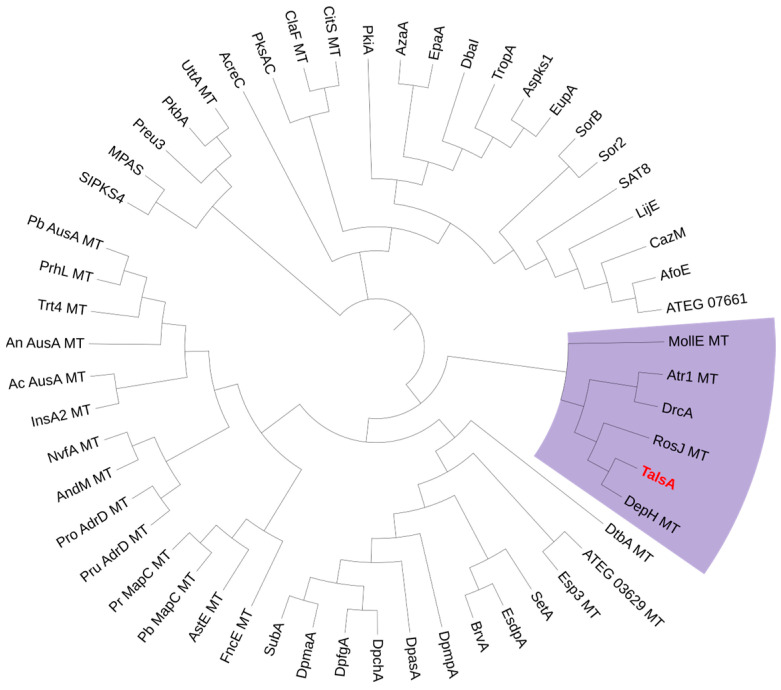
Phylogenetic analysis of the known fungal nonreducing polyketide synthases along with TalsA.

To investigate the secondary metabolic potential of *Talaromyces* sp. HDN1820200, its whole genome sequencing was performed. The prediction of potential BGC using antiSMASH indicated 25 PKSs, 20 NRPSs, 8 terpenes, 6 hybrids and 2 other types of BGCs (Figure S1). The nonreducing polyketide synthase (TalsA) which was located in BGC-clu14.1 shows moderate similarity (46%) with lecanoric acid synthase (Pre6) [7]. In addition, TalsA was located with six additional genes termed TalsBCDEFG, which was proposed to be P450 monooxygenase(P450), hypothetical protein (HP), FAD-dependent oxidoreductase (FAD-OR), decarboxylase and Diels–Alderase, respectively. Further analysis showed that only five genes were conserved in other fungi (Figure 4), which were nrPKS (TalsA), P450 (TalsB), FAD-OR (TalsE), decarboxylase (TalsF) and Diels–Alderase (TalsG). To our knowledge, such a BGC structure has never been reported yet. The further phylogenetic analysis of TalsA with other known NR-PKSs showed that it was located with depside synthases such as DrcA [8], Atr1 [9] and DepH [10] (Figure 5). These results strongly suggested that this BGC may be responsible for the biosynthesis of an unknown depside compound. The lack of a transcription factor in this gene cluster promoted us to investigate their functions by heterologous expression in *A. nidulans* A1145.

### 2.2. Heterologous Expression of the Tals Gene Cluster

**Figure 6:**
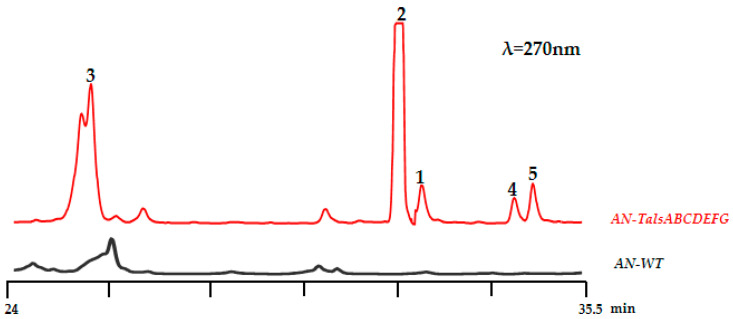
HPLC spectra of EtOAc crudes obtained from *A. nidulans* transformants *AN-TalsABCDEFG* with the native strain *A. nidulans* A1145.

To construct expression plasmids for the tals cluster (TalsA-G) in *A. nidulans*, each gene, along with its terminator, was cloned from the *Talaromyces* sp. HDN1820200 genome. Metabolite analysis showed that five additional peaks of compounds **1**–**5** were detected in the AN-talsABCDEFG EtOAc extract compared to that of the control strain containing the empty vector (Figure 6). The five compounds shared similar UV spectra with absorption maxima at 240, 280 and 330 nm, together with [M-H]^−^ ions at *m*/*z* = 345, 301, 181,345, and 359, respectively, indicating similar structures (Figure S12).

### 2.3. Structure Elucidation of Compounds **1**–**5**

Compounds **1**–**5** were isolated from the ethyl acetate extract of *AN-TalsA-G* cultures via repeated chromatographic purification (see Section 3.7 for details).

**Figure 7:**
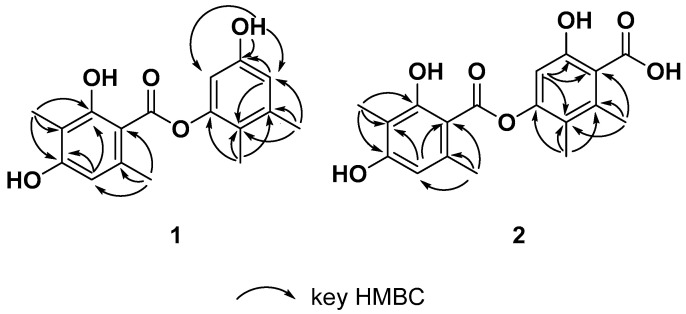
Key HMBC correlations of **1**–**2**.

Talaronic acid A (**1**) was obtained as an orange powder, and its molecular formula was established as C_17_H_18_O_5_ based on negative HRESIMS data, which displayed a peak at *m*/*z* 301.1084 (calculated for C_17_H_17_O_5_, [M-H]^−^, 301.1081). The ^1^H NMR spectrum of **1** showed ten proton signals, including four methyl protons, three exchangeable protons and three aromatic protons. (Table 1). The ^13^C NMR and HSQC spectrum showed twelve aromatic carbons for two phenyl units along with four methyl groups and only one carbonyl groups. The NMR data featured a depsidone-type derivative, which was structurally related to 4-*O*-demethylbarbatic acid [6]. Four methyl groups were directly connected to C-5(*δ*_C_ 118.3), C-6 (*δ*_C_ 138.6), C-3′ (*δ*_C_ 108.5) and C-6′ (*δ*_C_ 139.0) according to the HMBC correlations from H_3_-8 (*δ*_H_ 1.90) to C-4 (*δ*_C_ 149.9), C-5 and C-6, from H_3_-9 (*δ*_H_ 2.19) to C-1 (*δ*_C_ 106.7), C-5 and C-6, from H_3_-8′ (*δ*_H_ 1.96) to C-2′ (*δ*_C_ 162.6), C-3′ and C-4′ (*δ*_C_ 160.5), and lastly from H_3_-9′ (*δ*_H_ 2.49) to C-5′ (*δ*_C_ 110.9), C-6′ and C-1′ (*δ*_C_ 103.7). Moreover, the HMBC correlations from OH-2 (*δ*_H_ 9.39) to C-1, C-2 and C-3 (*δ*_C_ 114.8), from OH-2′ (*δ*_H_ 11.37) to C-1′ (*δ*_C_ 103.7), C-2′ and C-3′, and from OH-4′ (*δ*_H_ 10.30) to C-3′, C-4′ and C-5′ supported the location of three hydroxyl groups at C-2, C-2′ and C-4′. Furthermore, the HMBC correlations from H-1 (*δ*_H_ 6.40) to C-2 and C-5, from H-3 (*δ*_H_ 6.55) to C-1 and C-5, from H-5′ (*δ*_H_ 6.38) to C-1′, C-3′ and C-4′ and a weak HMBC correlation between H-5 (*δ*_H_ 6.38) and C-7′ completed the planar structure of **1** (Figure 7), which was given the trivial name talaronic acid A.

Talaronic acid B (**2**) was obtained as a purple powder, and its molecular formula was established as C_18_H_18_O_7_ based on negative HRESIMS data, which displayed a peak at *m*/*z* 345.0978 (calculated for C1_8_H_17_O_7_, [M-H]^−^, 345.0980). The ^1^H NMR spectrum of **2** showed six proton signals, including four methyl groups and two aromatic protons. (Table 1). The ^13^C NMR and HSQC spectrum showed twelve aromatic carbons for two phenyl units, four methyl groups and two carbonyl groups (Figures S2 and S3). The NMR data of **2** resembled those of compound **1** except for an additional carboxyl group in the ^13^C NMR spectra. Compound **2** was identified as a homologue of **1** with a carboxylic substitution at C-1 due to the disappearance of the HSQC signal at the H-1 position and the fact that the molecular weight of compound **2** was 346 on the basis of the HRESIMS data. A comparison of the ^13^C NMR spectrum of **1** and **2** showed that the chemical shift of C-4 is around 149 ppm and consisted of esterification-induced de-shielding, while that of C-2 is around 153 ppm, indicating no esterification at this position. This consists with literature examples where esterification also at C-4 results in a significant downfield shift (~5–10 ppm) due to increased electron withdrawal. Thus, the structure of compound **2** was determined and named talaronic acid B (**2**).

In addition, three known depsidones were obtained and proved to be identical to 3-methylorsellinic acid (**3**) [10], 4-*O*-demethylbarbatic acid (**4**) [5] and CJ-20557 (**5**) [11] based on the comparison of their spectroscopic data with those reported in the literature.

### 2.4. Testing of Anti-Inflammatory and Cytotoxic Activity

**Figure 8:**
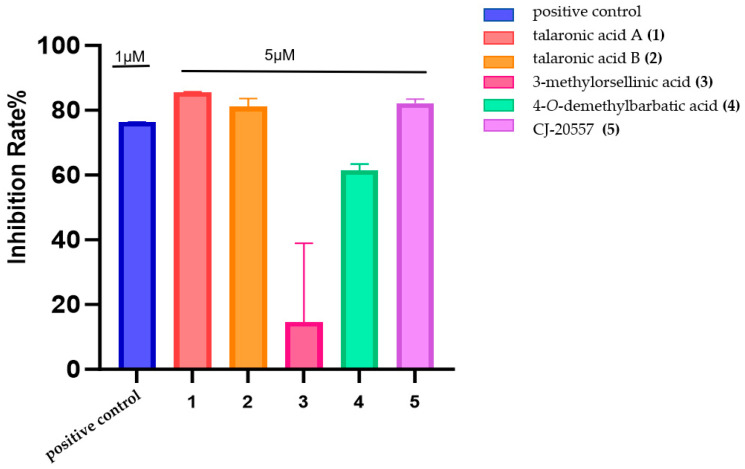
Anti-inflammatory activity of compounds **1**–**5**.

In the anti-inflammatory activity assay, talaronic acid A (**1**), talaronic acid B (**2**), and CJ-20557 (**5**) exhibited inhibition rates equivalent to the positive control (1 μM) at 5 μM (Figure 8). This observation aligns with previous studies on structurally related depsides. For example, physodic acid [12], perlatolic acid [13] and olivetoric acid [14] strongly suppressed mPGES-1 activity with IC_50_ values of 0.43, 0.4 and 1.15 μm, respectively. Similarly, MS-3, biosynthesized by *Stereum hirsutum*, exhibited noticeable NO inhibitory potential (IC_50_ 19.17 µM) in the LPS-induced macrophages compared with hydrocortisone (IC_50_ 48.15 µM) [15]. In addition, a trivaric acid, a *para*-tridepside, exhibited highly potent inhibitory activity against Human leukocyte elastase (HLE) with an IC_50_ value of 1.8 µM [16]. These results indicated the great potential of hetero-dimer depside as a promising source of novel bioactive agents.

In the cytotoxicity assay, compounds **1**–**5** were tested against K562 (human myeloid leukemia cells), L-02 (human normal liver cells), MDA-MB-231 (breast cancer cells), and ASPC-1 (human pancreatic cancer cells) for tumor cell growth inhibition activity. The results indicated that none of the compounds **1**–**5** displayed significant cytotoxic activity at 30 μM.

## 3. Materials and Methods

### 3.1. General Experimental Procedures

The instruments and main reagents used in this experiment are described as follows: SW-CJ series clean bench (Suzhou Antai Air Technology Co., Ltd., Suzhou, Jiangsu, China); CX41 biological microscope (CX41 biological microscope); Neofuge 15R/13R high-speed refrigerated centrifuge (Shanghai Lishen Scientific Instrument Co., Ltd., Shanghai, China); vacuum centrifugal concentrator and freeze dryer (CIMO International Group, Jinximo (Beijing) Co., Ltd., Beijing, China); LDZX-75KB vertical pressure steam sterilizer (Shanghai Shen’an Medical Equipment Factory, Shanghai, China); JA21002 electronic balance (Shanghai Jingtian Electronic Instrument Factory, Shanghai, China); SPX intelligent biochemical incubator (Ningbo Jiangnan Instrument Factory, Ningbo, Zhejiang, China); Life Pro gene amplification instrument (Hangzhou Bioer Technology Co., Ltd., Hangzhou, Zhejiang, China); SensiAnsys gel image analysis system (Shanghai Peiqing Technology Co., Ltd., Shanghai, China); EPS-600 electrophoresis apparatus (Shanghai Tianneng Technology Co., Ltd., Shanghai, China); HE-120 multifunctional horizontal electrophoresis tank (Shanghai Tianneng Technology Co., Ltd., Shanghai, China); gel recovery kit (OMEGA Co., Ltd., Norwalk, CT, USA); plasmid mini-prep kit (Tsingke Biotechnology Co., Ltd., Beijing, China); *E. coli* super competent cell preparation kit (Beyotime Biotechnology Co., Ltd., Shanghai, China); Hieff Canace^®^ high-fidelity DNA polymerase (Shanghai Yisheng Biotechnology Co., Ltd., Shanghai, China); 2×Hieff™ PCR Master Mix (With Dye) (Shanghai Yisheng Biotechnology Co., Ltd.); driselase (Shanghai Yuanye Biotechnology Co., Ltd., Shanghai, China); snailase (Shanghai Yuanye Biotechnology Co., Ltd., Shanghai, China); hygromycin (Solarbio Biotechnology Co., Ltd., Beijing, China); and ampicillin (Shanghai Sangon Biotech Co., Ltd., Shanghai, China). PCR primer synthesis and sequencing were performed by Sangon Biotech (Qingdao) Co., Ltd. (Qingdao, Shandong, China) The primers used in this study are listed in Table S2.

### 3.2. Materials and Culture Conditions

The marine sponge sample was collected by Tianjiao Zhu in Antarctic Weddell Sea specifically at coordinates 61°42′28″ S, 57°38′22″ W in the depth of 346 m on 11 January 2017. The sponge sample was identified as *Rossellinae* sp., and it has been deposited at the Marine Medicinal Bioresources Center (MMBC, Catalog No. MMBC-D1-4-6), Ocean University of China. The fungal strain *Talaromyces* sp. HDN1820200 was isolated from the sponge sample. The strain was identified by an internal transcribed spacer (ITS) sequence, and the sequence data were submitted to GenBank (GenBank accession no. MW031818). The strain was deposited at the Marine Medicinal Bioresources Center (MMBC), Ocean University of China, China. For genomic DNA extraction, *Talaromyces* sp. HDN1820200 was cultured at 28 °C on PDA plates for 5 days. *Escherichia coli* XL-1 was used for plasmids preservation and amplification. *Saccharomyces cerevisiae* BJ5464-NpgA was used for in vivo DNA recombination for plasmids construction. *A. nidulans* A1145 was grown at 37 °C in CD (0.1% glucose, 0.5 *v*/*v*% 20× nitrate salts, 0.01 *v*/*v*% trace elements, and 2% agar for solid media) media for sporulation, CDS (0.1% glucose, 1.2 M D-sorbitol, 0.5 *v*/*v*% 20× nitrate salts, 0.01 *v*/*v*% trace elements, and 2% agar for solid media) to screen transformants or in CD-ST (2% starch, 2% casamino acids, 5 *v*/*v*% 20× nitrate salts, 0.1 *v*/*v*% trace elements) media for heterologous expression and compound production. All medias were prepared with appropriate supplements, including 10 mM uridine, 5 mM uracil and/or 0.5 µg/mL pyridoxine HCl and/or 2.5 µg/mL riboflavin, depending on the plasmids being transformed. For RNA isolation, AN-TalsABCDEFG was cultured in CD-ST at 28 °C, 220 rpm for 3.5 days.

### 3.3. Sequence Analysis of the TalsA Gene

The whole genome sequencing data were analyzed by antiSMASH [17]. The phylogenetic analysis was conducted with MEGA-X software (version 10.0.0) [18] with the amino acid sequences of TalsA and reported nrPKSs retrieved from the National Center for Biotechnology Information (NCBI) [19]. The conserved domain of the TalsA protein was scanned by the InterProScan program [20]. Comparative analysis between the gene cluster and other homologous BGCs was conducted by Clinker [21].

### 3.4. Gene Cloning, Plasmid Construction, and Genetic Manipulation

As outlined in Table S1, the plasmids utilized in this study are enumerated. In addition, Table S2 details the oligonucleotide sequences of the PCR primers. The Q5^®^ High-Fidelity DNA polymerase and restriction endonuclease, indispensable for all DNA processing, were obtained from New England Biolabs (NEB). The Frozen-EZ Yeast Transformation II Kit (Zymo Research, Orange County, CA, USA) and the Zymoprep Yeast Plasmid Miniprep I Kit (Zymo Research) were employed for yeast transformation and plasmid recombination.

The expression plasmids were generated through yeast homologous recombination in *S. cerevisiae* BJ5464-NpgA [22]. The glaA, amyB, and gpdA promoters were amplified from plasmids pANU, pANR, and pANP, respectively, using the primer pairs glaA-F/R, amyB-F/R, and gpdA-F/R. Plasmid pANU was digested with NotI, while plasmids pANR and pANP were digested with BamHI to serve as vectors for gene insertion. To construct plasmid pANU-TalsA, TalsA was amplified by PCR using the primer pair pANU- TalsA -F/R and cloned into vector pANU.

For plasmid pANR-TalsBCD, TalsB, TalsC and TalsD were amplified using the primer pair pANR-TalsB-F/R, pANR-TalsC-F/R, and pANR-TalsD-F/R and cloned into vector pANR. The construction of plasmid pANP-TalsEFG involved the amplification of TalsE, TalsF and TalsG by PCR using the pANP-TalsE -F/R, pANP-TalsF -F/R, and pANG- TalsG -F/R. The obtained construct, pANU-TalsA, pANR-TalsBCD, and pANP-TalsEFG was introduced into *A. nidulans* A1145 by polyethylene glycol (PEG)-mediated protoplast transformation [23]. Integration transformants, including TalsA-G, were grown on solid CD–starch medium following selection by uridine and uracil, riboflavin and pyridoxine autotrophy and subsequent confirmation by PCR amplification. The cultures were extracted with an equal volume ethyl acetate and analyzed by LC-MS for secondary metabolites.

### 3.5. Transformation of A. nidulans A1145

*Aspergillus nidulans* A1145 was employed as the heterologous host. The preparation and transformation of fungal protoplasts were carried out according to the method described by Yee and Tang [24]. Three fungal transformants, namely *AN-TalsA*, *AN-TalsABCD*, and *AN-TalsABCDEFG*, were constructed using polyethylene glycol (PEG)-mediated protoplast transformation. Transformations with the empty vectors pANU, pANR, and pANP were performed as controls (*AN-WT*). The transformants were verified by PCR.

### 3.6. Fermentation and HPLC/LC-MS Analyses

The obtained transformants were cultured on solid CD-ST medium (10 g/L acid-hydrolyzed casein, 20 g/L starch, 1 mL/L trace elements, 50 mL/L nitrate, 20 g/L agar) and incubated at 28 °C for 5 days. Subsequently, the cultures were extracted three times with ethyl acetate (EtOAc). The organic phase was evaporated to dryness using a rotary evaporator and redissolved in 300 µL of methanol. Then, 50 µL of the dissolved extract was subjected to high-performance liquid chromatography–photodiode array detection-mass spectrometry (HPLC-DAD-MS) analysis (C18 column, Shimadzu (Japan), 4.6 mm × 150 mm, 5 µm, 1 mL/min). The samples were separated using a linear gradient of 15–50% CH_3_CN in water (0.1% trifluoroacetic acid) over 25 min at a flow rate of 1 mL/min, followed by a linear gradient of 50–100% CH_3_CN in water (0.1% trifluoroacetic acid) for 10 min, and finally isocratic elution with 100% CH_3_CN for 5 min. For HPLC analysis (C18 column, Shimadzu, 4.6 mm × 150 mm, 5 µm, 1 mL/min), the samples were first separated isocratically with 5% methanol for 5 min at a flow rate of 1 mL/min, followed by a linear gradient of 5–100% methanol (MeOH) in water (0.1% trifluoroacetic acid) over 30 min at a flow rate of 1 mL/min, and finally isocratic elution with 100% methanol for 5 min.

### 3.7. Extraction, Isolation, and Purification

The heterologous transformant *AN-TalsA-G* was subjected to large-scale cultivation in 5 L of CDST medium and incubated at 28 °C for 5 days. The solid medium obtained from the large-scale fermentation was extracted with ethyl acetate three times, yielding a crude extract (10 g). The crude extract was then fractionated using an ODS reverse-phase column with a gradient elution of MeOH/H_2_O, resulting in 10 fractions (Fr.1–Fr.10, 10–100%). Fr.4 was further purified on a preparative C18 HPLC column using an isocratic elution of MeOH/H_2_O (45:55), yielding compound **3** (3.2 mg). Fr.6 was purified on a preparative C18 HPLC column with an isocratic elution of MeOH/H_2_O (68:32), yielding compound **1** (3.6 mg) and compound **2** (3.4 mg). Fr.7 was purified on a preparative C18 HPLC column with an isocratic elution of MeOH/H_2_O (71:29), yielding compound **4** (3.3 mg) and compound **5** (3.7 mg).

Talaronic acid A (**1**): orange amorphous solid powder; UV (MeOH) λmax (log ε): 220(1.5), 275(0.8), ^1^H and ^13^C NMR data in Table 1; HRESIMS *m*/*z* 301.1084 [M-H]^−^ (calcd for C_17_H_17_O_5_ 301.1081).

Talaronic acid B (**2**): purple amorphous solid powder; UV (MeOH) λmax (log ε): 215(1.6), 275(0.8), ^1^H and ^13^C NMR data in Table 1; HRESIMS *m*/*z* 345.0978 [M-H]^−^ (calcd for C_18_H_17_O_7_ 345.0980).

### 3.8. Anti-Inflammatory and Cytotoxicity Assays

J774A.1 cells were seeded overnight in 96-well plates at a density of 5 × 10^5^ cells/mL. The cells were stimulated with LPS at a final concentration of 1 μg/mL for 4.5 h. Subsequently, the test compounds were added and incubated for 30 min, which was followed by the addition of ATP (10 mM) to induce NLRP3 inflammasome activation for 30 min. The supernatants were collected, and the levels of IL-1β were measured using a mouse IL-1β enzyme-linked immunosorbent assay (ELISA) kit according to the manufacturer’s instructions [25]. The cytotoxic activities of the five obtained compounds were evaluated against various cancer cell lines. The SRB assay was used to assess cytotoxicity against MDA-MB-231 [26], ASPC-1 [27], and L-02 [28] cell lines, while the MTT assay was employed for the K562 [29] cell line.

## 4. Conclusions

Bioinformatic analysis of *Talaromyces* sp. HDN1820200 revealed a putative secondary metabolite gene cluster potentially responsible for the biosynthesis of novel depside-like compounds. Through a heterologous expression strategy, the Tals gene cluster was successfully activated, leading to the isolation of two new depside-like compounds from the fermentation products. These compounds represent the first reported heterodimers of 3-MOA and 5-MOA. Bioactivity assays demonstrated that compounds **1** and **2** exhibit moderate anti-inflammatory activities. These findings have expanded the structural diversity of depside-like natural products and provide a valuable reference for the discovery of novel depside-like compounds.

## Figures and Tables

**Table 1:** ^1^H (500 MHz) and ^13^C (150 MHz) NMR spectroscopic data for compound **1** and **2** in DMSO-*d6*.

NO.	1		2	
	*δ*_C_	*δ*_H_ (*J* in Hz)	*δ*_C_	*δ*_H_ (*J* in Hz)
1	106.7, C	6.40, d (2.47)	121.9, CH	-
2	155.6, C	-	152.7, C	-
3	114.8, CH	6.55, d (2.46)	107.2, CH	6.55, s
4	149.9, C	-	149.0, C	-
5	118.3, C	-	119.1, C	-
6	138.6, C	-	135.3, C	-
7	-	-	169.6, C	-
8-CH_3_	11.7, CH_3_	1.90, s	11.9, CH_3_	1.94, s
9-CH_3_	19.9, CH_3_	2.19, s	16.9, CH_3_	2.19, s
2-OH	-	9.39, s	-	-
1′	103.7, C	-	103.8, C	-
2′	162.6, C	-	161.9, C	-
3′	108.5, C	-	108.6, C	-
4′	160.5, C	-	160.7, C	-
5′	110.9, CH	6.38, s	110.1, CH	6.39, s
6′	139.0, C	-	138.9, C	-
7′	169.9, C	-	169.0, C	-
8′-CH_3_	8.1, CH_3_	1.96, s	8.1, CH_3_	1.97, s
9′-CH_3_	23.5, CH_3_	2.49, s	23.7, CH_3_	2.48, s
2′-OH	-	11.37, s	-	11.25, s
4′-OH	-	10.30, s	-	10.36, s

## Data Availability

The data presented in this study are available in this article and the Supplementary Materials.

## Supplementary Materials

The following supporting information can be downloaded at https://www.mdpi.com/article/10.3390/md23030130/s1, Table S1. Strains and plasmids used in this study; Table S2. The primers used in this study; Table S3. Annotation of each gene in the Tals cluster from *Talaromyces* sp. HDN1820200; Table S4. ^1^H and ^13^C NMR spectroscopic data for compound **3** in DMSO-*d6* (150 and 400 MHz); Table S5. ^1^H and ^13^C NMR spectroscopic data for compound **4** in DMSO-*d6* (150 and 500 MHz); Table S6. ^1^H and ^13^C NMR spectroscopic data for compound **5** in DMSO-*d6* (100 and 500 Mz); Figure S1. AntiSMASH analysis of the *Talaromyces* sp. HDN1820200 genome. The predicted 61 gene clusters included 25 PKS, 20 NRPS, 8 terpenes, 6 PKS-NRPS hybrids, 1 siderophore, and 1 other gene cluster; Figure S2. Plasmid profiles constructed in this experiment pANU-TalsA, pANR-TalsBCD, pANP-TalsEFG; Figure S3. Results of PCR validation of plasmids constructed in this section pANU-TalsA, pANR-TalsBCD, pANP-TalsEFG; Figure S4. Sequencing validation results of plasmids pANU-TalsA, pANR-TalsBCD, pANP-TalsEFG constructed in this section; Figure S5. Protein sequence alignment of TalsA with other homologs using Blastp (protein–protein BLAST); Figure S6. Protein sequence alignment of TalsB with other homologs using Blastp (protein–protein BLAST); Figure S7. Protein sequence alignment of TalsC with other homologs using Blastp (protein–protein BLAST); Figure S8. Protein sequence alignment of Tals D with other homologs using Blastp (protein–protein BLAST); Figure S9. Protein sequence alignment of TalsE with other homologs using Blastp (protein–protein BLAST); Figure S10. Protein sequence alignment of TalsF with other homologs using Blastp (protein–protein BLAST); Figure S11. Protein sequence alignment of TalsG with other homologs using Blastp (protein–protein BLAST); Figure S12. LC-MS spectra of compounds isolated and purified from ethyl acetate extracts obtained from *A. nidulans* transformants at the same molecular weight of the native strain of *A. nidulans*; Figure S13. UV-vis spectra of purified compounds. All spectra were measured by an Agilent HPLC system equipped with a variable wavelength detector; Figure S14. ^1^H NMR of **1** in DMSO-*d6*; Figure S15. ^13^C NMR of **1** in DMSO-*d6*; Figure S16. COSY of **1** in DMSO-*d6*; Figure S17. HMBC of **1** in DMSO-*d6*; Figure S18. HSQC of **1** in DMSO-*d6*; Figure S19. HRESIMS of **1**; Figure S20. ^1^H NMR of **2** in DMSO-*d6*; Figure S21. ^13^C NMR of **2** in DMSO-*d6*; Figure S22. COSY of **2** in DMSO-*d6*.; Figure S23. HMBC of **2** in DMSO-*d6*; Figure S24. HSQC of **2** in DMSO-*d6*; Figure S25. HRESIMS of **2**; Figure S26.**^1^**H NMR of **3** in DMSO-*d6*; Figure S27.**^1^**H NMR of **4** in DMSO-*d6*; Figure S28.**^1^**H NMR of **5** in DMSO-*d6*; Figure S29. Picture of the isolation source of *Talaromyces* sp. HDN1820200.
