# Supplementary material for: Genome Mining-Guided Discovery of Two New Depsides from Talaromyces sp. HDN1820200
Source: Mar Drugs. 2025 Mar 18;23(3):130. doi: 10.3390/md23030130 (PMC11944171; doi:10.3390/md23030130)
Supplement: Supplementary file 1 [file marinedrugs-23-00130-s001.zip › marinedrugs-3530155-supplementary.pdf]

Contents

Table S1. Strains and plasmids used in this study 3
Table S2. The primers used in this study..4
Table S3. Annotation of each gene in the Tals cluster from *Talaromyces* sp. HDN1820200..........6
Table S4. ^{1}H and ^{13}C NMR Spectroscopic Data for Compounds **3** in DMSO-*d6* (150 and 400 MHz). 7
Table S5. ^{1}H and ^{13}C NMR Spectroscopic Data for Compounds **4** in DMSO-*d6* (150 and 500 MHz). 8
Table S6. ^{1}H and ^{13}C NMR Spectroscopic Data for Compounds **5** in DMSO-*d6* (100 and 500 MHz) 9
Figure S1. AntiSMASH analysis of the *Talaromyces* sp. HDN1820200 genome. The predicted 61 gene clusters included 25 PKS, 20 NRPS, 8 terpenes, 6 PKS-NRPS hybrids, 1 siderophore, and 1 other gene cluster...10
Figure S2. Plasmid profiles constructed in this experiment pANU-TalsA,pANR-TalsBCD,pANP-TalsEFG 11
Figure S3. Results of PCR validation of plasmids constructed in this section pANU-TalsA, pANR-TalsBCD, pANP-TalsEFG...12
Figure S4 Sequencing validation results of plasmids pANU-TalsA, pANR-TalsBCD, pANP-TalsEFG constructed in this experiment 13
Figure S5. Protein sequence alignment of TalsA with other homologs using Blastp (protein-protein BLAST)...14
Figure S6. Protein sequence alignment of TalsB with other homologs using Blastp (protein-protein BLAST)...15
Figure S7. Protein sequence alignment of TalsC with other homologs using Blastp (protein-protein BLAST)...16
Figure S8. Protein sequence alignment of TalsD with other homologs using Blastp (protein-protein BLAST)...17
Figure S9. Protein sequence alignment of TalsE with other homologs using Blastp (protein-protein BLAST)...18
Figure S10. Protein sequence alignment of TalsF with other homologs using Blastp (protein-protein BLAST)...19
Figure S11. Protein sequence alignment of TalsG with other homologs using Blastp (protein-protein BLAST)...20
Figure S12. LC-MS spectra of compounds isolated and purified from ethyl acetate extracts obtained from A. nidulans transformants against the same molecular weight of the native strain of A. nidulans...21
Figure S13. UV-Vis spectra of purified compounds. All spectra were measured by an Agilent HPLC system equipped with a variable wavelength detector..22
Figure S14. ^{1}H NMR of **1** in DMSO-*d6*..23
Figure S15. ^{13}C NMR of **1** in DMSO-*d6*...23
Figure S16. COSY of **1** in DMSO-*d6*...24
Figure S17. HMBC of **1** in DMSO-*d6*...24

Figure S18. HSQC of **1** in DMSO-*d6*...25
Figure S19. HRESIMS of **1**...25
Figure S20. ^{1}H NMR of **2** in DMSO-*d6*...26
Figure S21. ^{13}C NMR of **2** in DMSO-*d6*...26
Figure S22. COSY of **2** in DMSO-*d6*...27
Figure S23. HMBC of **2** in DMSO-*d6*...27
Figure S24. HSQC of **2** in DMSO-*d6*...28
Figure S25. HRESIMS of **2**...28
Figure S26. ^{1}H NMR of **3** in DMSO-*d6*...29
Figure S27. ^{1}H NMR of **4** in DMSO-*d6*...29
Figure S28. ^{1}H NMR of **5** in DMSO-*d6*...30
Figure S29. Picture of the isolation source of *Talaromyces* sp. HDN1820200...30

Table S1. Strains and plasmids used in this study

Strain/Plasmid	Characteristics/Use	source
**strains**		
Talaromyces sp. HDN1820200	isolated from Antarctic sponge samples	This study
Escherichia coli XL-1	Host bacteria for plasmid preservation and cloning	This study
Saccharomy cescerevisiae BJ5464-NpgA	Nutritionally deficient yeast strains (Ura-, Trp-, Leu-) as vectors with homologous recombination of gene fragments in host strains	This study
Aspergillus.nidulans A1145	Nutrient-deficient Aspergillus oryzae (Ura-/Uri-, Riboflavin-, Pyridoxine-), as a host strain for gene heterologous expression	This study
AN-empty vectors	Harboring three empty pANU, pANR, and pANP vectors	This study
AN-Tals A	Harboring the plasmid pANU-Tals A	This study
AN-Tals ABCD	Harboring the plasmid pANU-TalsA and pANR-BCD	This study
AN-TalsABCDEFG	Harboring the plasmid pANU-TalsA and pANR-BCD and pANP-EFG	This study
**plasmids**		
pANU	Contains the UraC backfill gene as an Aspergillus nidulans heterologous expression vector plasmid with the gene fragment inserted after the promoter glaA	This study
pANR	Containing the riboB backfill gene as a heterologous expression vector plasmid for Aspergillus constructus, the gene fragment was inserted after the promoter gpdA-5′ promoter	This study
pANP	Containing the pyroA backfill gene as a heterologous expression vector plasmid for Aspergillus constructus, the gene fragment was inserted after the initiation of the AmyB-5′	This study
pANU-TalsA	TalsA gDNA with downstream 400 bp used the promoter glaA in PacI-linearized pANU	This study
pANR-TalsBCD	TalsB gDNA with downstream 300 bp, TalsC gDNA with downstream 300 bp and TalsD gDNA with downstream 300 bp in PacI-linearized pANR	This study
pANP-TalsEFG	TalsE gDNA with downstream 300 bp, TalsF gDNA with downstream 300 bp and TalsG gDNA with downstream 500 bp in PacI-linearized pANR	This study

Table S2. The primers used in this study

Primer name	sequences（5′→3′）	application
pANU-*TalsA*-F	GCCTGAGCTTCATCCCCAGCATCATTACACCTCAGCATCACGCAATAATGGGCGAGCAG	For TalsA fragment amplification and recombinant plasmid validation
pANU-*TalsA*-R	GTGGAGGACATACCCGTAATTTTCTGGGCATTTAAATCGCAAACAGGCCTATCCTGATC	
pANR-*TalsB*-F	aaccattaccccgccacatagacacatctaaacaCCAGCACACCATGAATAATAACACC	For TalsB fragment amplification and recombinant plasmid validation
pYTR-*TalsB*-R	TAAAGGGTATCATCGAAAGGGAGTCATCCAATTTAAATACCTTTCACCTCTTTGGCTGG	
pANR-*TalsC*-F	GAGCTTCATCCCCAGCATCATTACACCTCAGCAATGTTGCTCCTTCCAGGC	For TalsC fragment amplification and recombinant plasmid validation
pYTR-*TalsC*-R	CGCTAGATCTAGTGTAAAGTTGGGGATTAAAGGTGCCGAACGAGCTATAAATGATATAA	
pANR-*TalsD*-F	CCTTCTCTGAACAATAAACCCCACAGAAGGCATTTATGGTGCTCAAGTTTAGTCATCTA	For TalsD fragment amplification and recombinant plasmid validation
pYTR-*TalsD*-R	CTTTCCCCCCTCCTCTTAATTATTGATGTGCCCTAATCATATGCCCATTTAAATTGGAT	
pANP-*TalsE*-F	TCTCCCTTCTCTGAACAATAAACCCCACAGAAGGCATTTATGGCGCCTCCCAGGATTGA	For TalsE fragment amplification and recombinant plasmid validation
pANP-*TalsE*-R	ATAGGTCGCCAGGTACGACCAGTTCGGAAGATCAGGCTAGAAGCTCTGTGGAAGCAGAC	
pANP-*TalsF*-F	GAGCCTGAGCTTCATCCCCAGCATCATTACACCTCAGCAGCAATAGCAATGGCCGCCAC	For TalsF fragment amplification and recombinant plasmid validation
pANP-*TalsF*-R	gggtctctcccgtcacccaaatcaattcaccggagtGAGCGATTTGTTGCGTTATGGTG	
pANP-*TalsG*-F	tgactaaccattaccccgccacatagacacatctaaacaATGCGCAGTCTTGTCGCATC	For TalsG fragment amplification and recombinant plasmid validation
pANP-*TalsG*-R	ATGAGACCCAACAACCATGATACCAGGGGATTTAAATGATGGTGAGGAGACGAGGCAGA	

GPDA-F	actccggtgaattgatttgggtg	For amplification of GPDA gene fragments
GPDA-R	tgtttagatgtgtctatgtggcgg	
PGLA-F	CCTGATCTTCCGAACTGGTCG	For amplification of PGLA gene fragments
PGLA-R	TGCTGAGGTGTAATGATGCTGG	
Amyb-F	GATTAAAGGTGCCGAACG	For amplification of Amyb gene fragments
Amyb-R	ACCCCACAGAAGGCATTT	

Table S3. Annotation of each gene in the Tals cluster from *Talaromyces* sp. HDN1820200

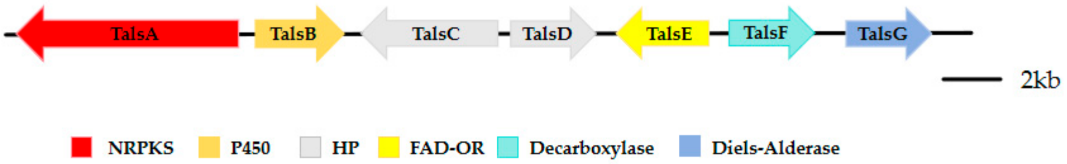

Genes	Size	Translate	Putative Function	Closest Homolog (Origin, Accession No.)	Identity (%)
TalsA	2575	-	NR-PKS	Preussia isomera P9WET2.1	2197/2575 (46.63%)
TalsB	514	+	P450	Hormonema carpetanum A0A2Z4HPZ7.1	505/514(35.57%)
TalsC	863	-	HP	-	-
TalsD	433	+	HP	-	-
TalsE	499	-	FAD-OR	Aspergillus niger ATCC 1015 G3XMD0.2	472/499(38.22%)
TalsF	319	+	Decarboxylase	Penicillium terrestre A0A2P1DP90.1	338/319(43.03%)
TalsG	321	+	Diels-Alderase	Sarocladium sp. 'schorii' A0A2U8U2M1.1	401/321(23.91%)

Table S4.^1H and ^{13}C NMR Spectroscopic Data for Compound **3** in DMSO-*d6* (150 and 400 MHz).

NO.	δ_C	δ_H(*J* in Hz)
1	103.5, C	-
2	163.1, C	-
3	107.9, C	-
4	159.9, C	-
5	110.3, CH	6.24, s
6	139.6, C	-
7	174.2, C	-
3′	8.0, CH_3	1.97, s
6′	23.8, CH_3	1.96, s
2-OH	-	11.26, s
4-OH	-	10.33, s

Table S5. ^{1}H and ^{13}C NMR Spectroscopic Data for Compound **4** in DMSO-*d6* (150 and 500 MHz).

NO.	δC	δH(*J* in Hz)
1	112.0, C	-
2	162.0, C	-
3	114.6, C	-
4	152.0, C	-
5	115.6, CH	6.63, s
6	139.0, C	-
7	173.1, C	-
8	9.2, CH_3	1.97-1.98, d(5.40)
9	22.9, CH_3	2.45-2.48, s
1′	103.7, C	-
2′	161.0, C	-
3′	108.6, C	-
4′	160.8, C	-
5′	110.9, CH	6.39, s
6′	139.0, C	-
7′	169.3, C	-
3′-CH3	8.1, CH_3	1.94-1.98, d(5.40)
6′-CH3	23.6, CH_3	2.45-2,48, s
2′-OH	-	11.26, s
4′-OH	-	10.33, s

Table S6. ^{1}H and ^{13}C NMR Spectroscopic Data for Compound **5** in DMSO-*d6* (100 and 500 Mz)

NO.	δC	δH(*J* in Hz)
1	118.5, C	-
2	154.0, C	-
3	116.3, C	-
4	148.9, C	-
5	119.6, C	-
6	134.0, C	-
7	171.2, C	-
8	9.8, CH_3	1.95, s
9	12.6, CH_3	1.96, s
10	17.4, CH_3	2.31, s
1′	103.6, C	-
2′	162.7, C	-
3′	108.0, C	-
4′	160.7, C	-
5′	110.6, CH	6.41, s
6′	140.3, C	-
7′	168.9, C	-
8′	8.0, CH_3	1.97, s
9′	24.0, CH_3	2.53, s
2′-OH	-	11.26, s
4′-OH	-	10.33, s

Select genomic region:

Overview 1.1 1.2 1.3 1.4 2.1 2.2 2.3 2.4 3.1 3.2 3.3 4.1 4.2 4.3 4.4 6.1 6.2 7.1 8.1 8.2 9.1 10.1 11.1 11.2 12.1 13.1 14.1 16.1 16.2 17.1 18.1 18.2 18.3 19.1 20.1 21.1 22.1 22.2 22.3 25.1 25.2 25.3 26.1 29.1 31.1 32.1 33.1 34.1 35.1 36.1 36.2 39.1 41.1 41.2 47.1 51.1 59.1 63.1 66.1 71.1 92.1

Identified secondary metabolite regions using strictness 'relaxed'

Region	Type	From	To	Most similar known cluster		Similarity
Region 1.1	T1PKS	466,431	508,726	sorbicillin	Polyketide	28%
Region 1.2	T1PKS , NRPS , NRPS-like	563,103	675,730	ilicicolin H	NRP + Polyketide	83%
Region 1.3	T1PKS	695,094	742,505			
Region 1.4	NRPS	1,274,584	1,347,274	cyclochlorotine / hydroxy-cyclochlorotine / cyclotine / cyclochlorotine B / deoxy-Cyclochlorotine	NRP	37%
Region 2.1	T1PKS	28,210	74,269			
Region 2.2	NRPS	166,510	232,872			
Region 2.3	T1PKS	340,146	408,563			
Region 2.4	T1PKS , NRPS	784,957	839,739	wortmanamide A / wortmanamide B	NRP + Polyketide	33%
Region 3.1	NRPS-like	540,948	584,411			
Region 3.2	terpene	683,113	704,689	squalestatin S1	Terpene	40%
Region 3.3	T1PKS	836,509	881,991	endocrocin	Polyketide:Iterative type I	22%
Region 4.1	NRPS-like	171,242	214,379			
Region 4.2	NRPS-like	226,930	270,894			
Region 4.3	T1PKS	504,370	551,181	naphthopyrone	Polyketide	100%
Region 4.4	terpene	969,915	991,512			
Region 6.1	T1PKS	89,923	137,891			
Region 6.2	NRPS-like	1,065,532	1,103,322			
Region 7.1	NRPS	813,661	867,901			
Region 8.1	T1PKS	133,815	180,896			
Region 8.2	terpene	241,219	265,345			
Region 9.1	siderophore	496,749	510,208			
Region 10.1	T1PKS	648,543	696,947			
Region 11.1	T1PKS	147,290	194,827	viridicatumtoxin / previridicatumtoxin / 5-hydroxyanthrotainin / 8-O-desmethylanthrotainin	Polyketide:Iterative type I	13%
Region 11.2	NRPS-like	212,338	255,750			
Region 12.1	T1PKS	59,805	109,158	betaenone C / betaenone A	Polyketide	37%
Region 13.1	T1PKS , NRPS-like	222,696	308,752	swainsonine	Polyketide	100%
Region 14.1	T1PKS	400,454	448,452	chrysoxanthone A / chrysoxanthone B / chrysoxanthone C	Polyketide	8%
Region 16.1	T1PKS	97,464	137,368			
Region 16.2	T1PKS	548,806	597,246			
Region 17.1	NRPS-like	71,966	115,118			
Region 18.1	terpene	137,899	160,655	asperterpenoid A	Terpene	100%
Region 18.2	NRPS	438,587	481,651			
Region 18.3	T1PKS , NRPS	552,853	605,299	curvupallide-B	NRP + Polyketide	11%
Region 19.1	T1PKS	450,721	516,215	ankaflavin / monascin / rubropunctatine / monascorubrin	Polyketide:Iterative type I	12%
Region 20.1	NRPS-like	20,602	64,518			
Region 21.1	T1PKS	409,727	454,897			
Region 22.1	NRPS	5,033	59,216			
Region 22.2	terpene	87,644	108,681			
Region 22.3	T1PKS	334,679	382,451	phomoidride	Polyketide	53%
Region 25.1	betalactone	1	25,859			
Region 25.2	terpene	170,027	191,357	copalyl diphosphate	Terpene	28%
Region 25.3	other	256,357	297,292			
Region 26.1	terpene	132,321	153,573			
Region 29.1	T1PKS	114,004	160,617	naphthopyrone	Polyketide	100%
Region 31.1	T1PKS	7,783	55,359			
Region 32.1	NRPS	241,319	301,186	aspercryptins	NRP	20%
Region 33.1	NRPS	85,112	144,605			
Region 34.1	T1PKS	196,758	244,815			
Region 35.1	NRPS-like	205,944	249,320			
Region 36.1	NRPS-like	2,459	41,255			
Region 36.2	NRPS	129,865	194,070			
Region 39.1	T1PKS , NRPS-like	14,631	74,591	cichorine	Polyketide	100%
Region 41.1	T1PKS	488	42,411			
Region 41.2	T1PKS	113,865	162,199			
Region 47.1	NRPS	71,351	126,834			
Region 51.1	T1PKS , NRPS-like	1	51,553			
Region 59.1	T1PKS	1	28,316			
Region 63.1	T1PKS	9,313	54,864	neosartorin	Polyketide	36%
Region 66.1	NRPS-like	31,097	56,996			
Region 71.1	NRPS-like	1	35,346			
Region 92.1	T1PKS	1	16,898			

Figure S1. AntiSMASH analysis of the *Talaromyces* sp. HDN1820200 genome. The predicted 61 gene clusters included 25 PKS, 20 NRPS, 8 terpenes, 6 PKS-NRPS hybrids, 1 siderophore, and 1 other gene cluster.

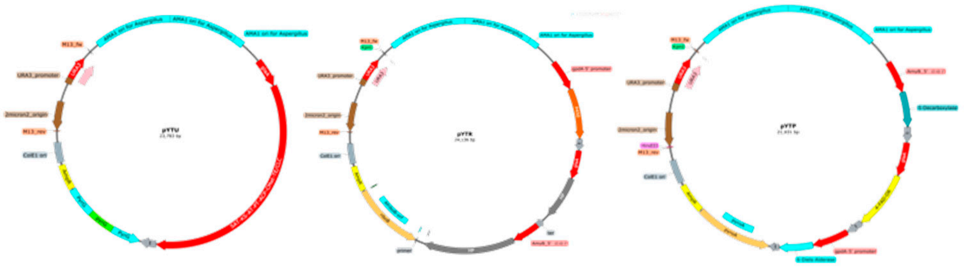

Figure S2. Plasmid profiles constructed in this experiment pANU-TalsA, pANR-TalsBCD, pANP-TalsEFG

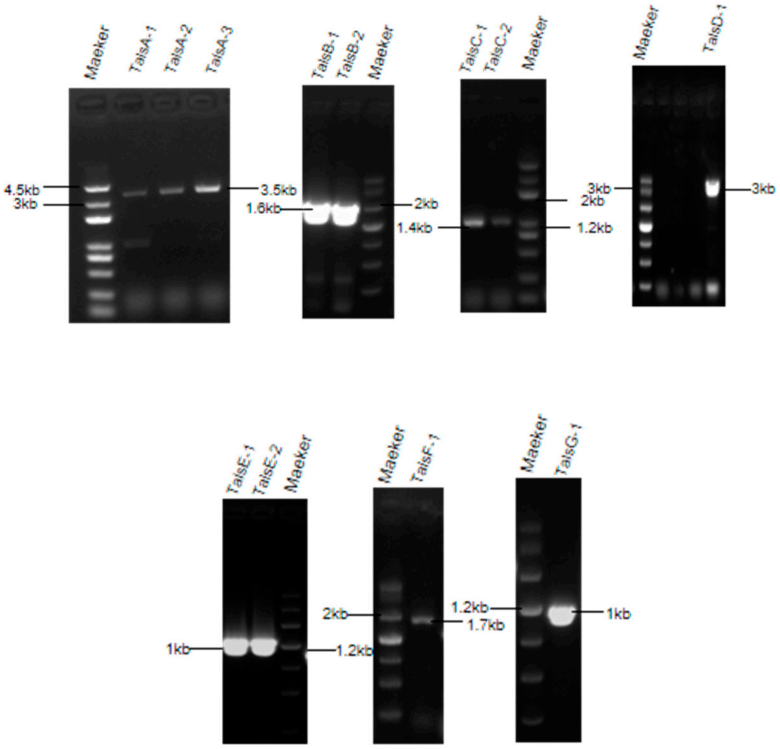

Figure S3. Results of PCR validation of plasmids constructed in this experiment pANU-TalsA, pANR-TalsBCD, pANP-TalsEFG

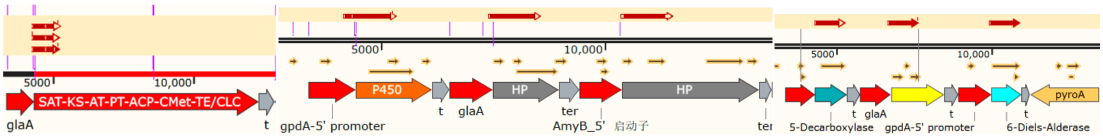

Figure S4 Sequencing validation results of plasmids pANU-TalsA, pANR-TalsBCD, pANP-TalsEFG constructed in this experiment

Description	Scientific Name	Max Score	Total Score	Query Cover	E value	Per. Ident	Acc. Len	Accession
RecName: Full=Non-reducing polyketide synthase Preu6; Short=NR-PKS Preu6; AltName: Full=Lecanoric acid ...	Preussia isomera	1675	2060	85%	0.0	46.63%	2197	P9WET2.1
RecName: Full=Non-reducing polyketide synthase atr1; AltName: Full=Atranorin biosynthesis cluster protein 1 [...	Stereocaulon alp...	1558	2274	99%	0.0	45.90%	2500	A0A8F4SKJ7.1
RecName: Full=Non-reducing polyketide synthase pks12; Short=NR-PKS pks12; AltName: Full=Mitorubrinol an...	Talaromyces mar...	1462	1462	68%	0.0	42.94%	1806	B6QK81.1
RecName: Full=5-methylorsellinic acid synthase; AltName: Full=Azasperpyranone A biosynthesis cluster A prot...	Aspergillus terre...	1447	1447	99%	0.0	34.66%	2590	Q0CRQ5.1
RecName: Full=Non-reducing polyketide synthase pkbA; Short=NR-PKS pkbA; AltName: Full=Cichorine biosynt...	Aspergillus nidul...	1379	1379	96%	0.0	34.19%	2517	Q5AZ32.1
RecName: Full=Non-reducing polyketide synthase ascC; AltName: Full=Ascofuranone/ascochlorin biosynthesis...	Acremonium egy...	1346	1683	80%	0.0	41.16%	2115	A0A455R5P9.1
RecName: Full=Non-reducing polyketide synthase stbA; AltName: Full=Ilicicolin B biosynthesis cluster protein s...	Stachybotrys bisbyi	1313	1630	84%	0.0	39.61%	2220	A0A193PS74.1
RecName: Full=Non-reducing polyketide synthase pigA; Short=nrPKS pigA; AltName: Full=Azaphilone pigment...	Monascus ruber	1138	1138	86%	0.0	32.13%	2690	A0A0S2CHA9.1
RecName: Full=Non-reducing polyketide synthase esdpA; Short=NR-PKS esdpA; AltName: Full=Shearone I bio...	Penicillium shearii	1114	1114	86%	0.0	32.69%	2193	A0A8D5M692.1
RecName: Full=Non-reducing polyketide synthase dpmpA; AltName: Full=Diterpenoid pyrone biosynthesis clust...	Macrophomina p...	1104	1104	85%	0.0	32.73%	2214	K2QVI8.1
RecName: Full=3-methylorcinaldehyde synthase tropA; Short=MOS; AltName: Full=Non-reducing polyketide sy...	Talaromyces stip...	1063	1063	84%	0.0	32.13%	2661	B8M9J9.1
RecName: Full=Non-reducing polyketide synthase dpchA; AltName: Full=Diterpenoid pyrone biosynthesis clust...	Colletotrichum hi...	1001	1001	86%	0.0	31.62%	2224	A0A1B7YCC9.1
RecName: Full=Non-reducing polyketide synthase dpasA; AltName: Full=Diterpenoid pyrone biosynthesis clust...	Apiospora sacchari	993	993	85%	0.0	31.00%	2159	P9WEX0.1
RecName: Full=Methylphloroacetophenone synthase; Short=MPAS; AltName: Full=Non-reducing polyketide sy...	Cladonia uncialis	903	1351	92%	0.0	33.85%	2545	A0A0R8YWJ7.2
RecName: Full=Citrinin polyketide synthase; Short=CitS; AltName: Full=Non-reducing polyketide synthase citS [...	Monascus ruber	891	1155	86%	0.0	32.42%	2593	A0A161CEU9.1
RecName: Full=Non-reducing polyketide synthase dpfgA; AltName: Full=Diterpenoid pyrone biosynthesis cluste...	Fusarium gramin...	887	1138	83%	0.0	33.06%	2172	I1RL11.1
RecName: Full=Non-reducing polyketide synthase dbaI; Short=NR-PKS dbaI; AltName: Full=Derivative of benz...	Aspergillus nidul...	876	1095	86%	0.0	31.89%	2605	Q5AUX7.2
RecName: Full=Non-reducing polyketide synthase Preu3; Short=NR-PKS Preu3; AltName: Full=3-methylorselli...	Preussia isomera	870	1301	97%	0.0	32.62%	2523	P9WET6.1
RecName: Full=Non-reducing polyketide synthase andM; AltName: Full=Anditomin synthesis protein M [Aspergi...	Aspergillus stella...	868	1348	98%	0.0	32.92%	2430	A0A097ZPE0.1
RecName: Full=Non-reducing polyketide synthase mapC; AltName: Full=Mycophenolic acid biosynthesis cluste...	Penicillium roque...	867	1328	93%	0.0	34.09%	2477	W6QL47.1
RecName: Full=Clavatol synthase claF; AltName: Full=Clavatol biosynthesis cluster protein F; AltName: Full=N...	Penicillium crust...	865	1086	87%	0.0	31.61%	2587	A0A481WQB6.
RecName: Full=Non-reducing polyketide synthase ausA; AltName: Full=Austinoid biosynthesis clusters protein ...	Penicillium brasil...	849	1328	94%	0.0	33.63%	2475	A0A0F7U103.1
RecName: Full=Non-reducing polyketide synthase nvfA; AltName: Full=Novofumigatonin biosynthesis cluster pr...	Aspergillus novof...	847	1257	93%	0.0	32.74%	2255	A0A2I1BSV9.1
RecName: Full=Non-reducing polyketide synthase pks11; Short=NR-PKS pks11; AltName: Full=Mitorubrinol an...	Talaromyces mar...	837	1033	84%	0.0	31.56%	2575	B6QK76.1
RecName: Full=Non-reducing polyketide synthase dpmaA; AltName: Full=Diterpenoid pyrone biosynthesis clust...	Metarhizium anis...	831	1082	77%	0.0	32.97%	2181	P9WEX6.1
RecName: Full=Non-reducing polyketide synthase prhL; AltName: Full=Paraherquonin biosynthesis cluster prot...	Penicillium brasil...	831	1295	95%	0.0	32.91%	2475	A0A1E1FFN8.1
RecName: Full=Non-reducing polyketide synthase mapC; AltName: Full=Mycophenolic acid biosynthesis cluste...	Penicillium brevi...	830	1318	95%	0.0	33.76%	2448	F1DBA9.1
RecName: Full=Non-reducing polyketide synthase subA; AltName: Full=Subglutinol biosynthesis cluster protein ...	Metarhizium rob...	826	1075	77%	0.0	33.03%	2181	E9F5E7.1
RecName: Full=Non-reducing polyketide synthase pkiA; Short=NR-PKS pkiA; AltName: Full=Pki biosynthesis cl...	Aspergillus nidul...	825	1007	82%	0.0	32.26%	2493	Q5B7U4.1
RecName: Full=Non-reducing polyketide synthase epaA; Short=NR-PKS epaA; AltName: Full=Pestalamide A bi...	Aspergillus niger...	825	1021	86%	0.0	30.90%	2617	A2QTE9.1
RecName: Full=Non-reducing polyketide synthase azaA; Short=NR-PKS azaA; AltName: Full=Azaphilone biosy...	Aspergillus niger ...	825	1030	86%	0.0	30.94%	2599	G3XMC4.1
RecName: Full=Non-reducing polyketide synthase adrD; AltName: Full=Andrastin A biosynthesis cluster protein...	Penicillium roque...	819	1299	100%	0.0	31.80%	2495	A0A1Y0BRF1.1
RecName: Full=Non-reducing polyketide synthase mapC'; AltName: Full=Mycophenolic acid biosynthesis cluste...	Penicillium brevi...	803	1040	77%	0.0	32.97%	2190	A0A0B5KU17.1
RecName: Full=Triacetic acid lactone synthase cle1; Short=TAL synthase cle1; AltName: Full=Chevalone E bio...	Aspergillus versi...	803	803	71%	0.0	30.89%	1807	A0A3T0ZHJ0.1
RecName: Full=Non-reducing polyketide synthase adrD; AltName: Full=Andrastin A biosynthesis cluster protein...	Penicillium ruben...	801	1281	99%	0.0	31.79%	2496	B6HV32.2
RecName: Full=Non-reducing polyketide synthase sor2; Short=NR-PKS sor2; AltName: Full=Sorbicillinoid biosy...	Trichoderma ree...	801	1027	88%	0.0	30.75%	2633	G0R6S9.1
RecName: Full=Non-reducing polyketide synthase SAT8; AltName: Full=Satratoxin biosynthesis SC1 cluster pr...	Stachybotrys ch...	800	991	82%	0.0	32.43%	2602	A0A084B9Z6.1
RecName: Full=Non-reducing polyketide synthase ausA; AltName: Full=Austinoid biosynthesis cluster protein A...	Aspergillus calid...	799	1286	95%	0.0	32.33%	2462	A0A0U5GFS8.1
RecName: Full=Non-reducing polyketide synthase ausA; AltName: Full=Austinoid biosynthesis clusters protein ...	Aspergillus nidul...	797	1264	98%	0.0	31.60%	2476	Q5ATJ7.1
RecName: Full=Iterative polyketide synthase CazM [Chaetomium globosum CBS 148.51]	Chaetomium glo...	797	964	79%	0.0	30.91%	2746	A0A0K0MCJ4.1
RecName: Full=Non-reducing polyketide synthase pkfA; Short=NR-PKS pfkA; AltName: Full=Aspernidine A bios...	Aspergillus nidul...	796	796	67%	0.0	31.39%	2193	Q5B8A0.1
RecName: Full=Non-reducing polyketide synthase ATEG_07661; Short=NR-PKS; AltName: Full=Azasperpyran...	Aspergillus terre...	795	967	79%	0.0	30.79%	2737	Q0CF73.1
RecName: Full=Non-reducing polyketide synthase sorB; Short=NR-PKS sorB; AltName: Full=Sorbicillinoid bios...	Penicillium ruben...	790	1013	86%	0.0	30.86%	2664	B6HN77.1
RecName: Full=Non-reducing polyketide synthase AC; Short=pksAC; AltName: Full=Ascochitine biosynthesis cl...	Ascochyta fabae	787	1004	85%	0.0	30.68%	2645	A0A5C1RD96.1
RecName: Full=Non-reducing polyketide synthase pkdA; Short=NR-PKS pkdA; AltName: Full=Pkd biosynthesis...	Aspergillus nidul...	776	930	81%	0.0	30.18%	2476	Q5BG07.1
RecName: Full=Iterative polyketide synthase afoE; AltName: Full=Asperfuranone biosynthesis protein B [Asper...	Aspergillus nidul...	761	931	78%	0.0	30.05%	2793	Q5BEJ6.1
RecName: Full=Non-reducing polyketide synthase trt4; AltName: Full=Terretonin synthesis protein 4 [Aspergillu...	Aspergillus terre...	744	1187	92%	0.0	31.73%	2422	Q0C8A4.2
RecName: Full=Non-reducing polyketide synthase pkhA; Short=NR-PKS pkhA; AltName: Full=Pkh biosynthesis...	Aspergillus nidul...	740	740	65%	0.0	28.48%	2221	Q5BBP8.1
RecName: Full=3-methylorcinaldehyde synthase; Short=MOS; AltName: Full=Non-reducing polyketide synthas...	Sarocladium sp. ...	736	907	83%	0.0	29.52%	2729	A5PHD6.2
RecName: Full=3-methylorcinaldehyde synthase; Short=MOS; AltName: Full=Eupenifeldin biosynthesis cluster ...	Phoma sp.	733	964	81%	0.0	30.14%	2682	A0A4P8GHJ1.1
RecName: Full=Non-reducing polyketide synthase tazA; Short=NR-PKS tazA; AltName: Full=Azaphilone biosyn...	Aspergillus terre...	668	907	85%	0.0	28.46%	2556	Q0CSA2.1
RecName: Full=Polyketide synthase 2 [Metarhizium majus ARSEF 297]	Metarhizium maj...	579	579	67%	1e-168	27.73%	2158	A0A0B4I1J8.1
RecName: Full=Polyketide synthase 1; AltName: Full=Conidial pigment biosynthesis polyketide synthase [Metar...	Metarhizium acri...	571	571	55%	1e-165	29.50%	2148	A0A2U8NEV6.1
RecName: Full=Polyketide synthase 2 [Metarhizium hybridum]	Metarhizium hyb...	570	570	67%	2e-165	27.42%	2155	A0A0B4FLB2.1
RecName: Full=Polyketide synthase 2 [Metarhizium acridum CQMa 102]	Metarhizium acri...	563	563	55%	7e-165	29.15%	1861	E9EHG2.1
RecName: Full=Orsellinic acid synthase armB; Short=OAS; AltName: Full=Melledonol synthase; AltName: Full=...	Armillaria gallica	569	569	66%	9e-165	28.48%	2209	A0A2H3CTK0.1
RecName: Full=Polyketide synthase 2 [Metarhizium guizhouense ARSEF 977]	Metarhizium guiz...	566	566	67%	4e-164	27.54%	2157	A0A0B4I1L1.1
RecName: Full=Orsellinic acid synthase ArmB; Short=OAS; AltName: Full=Melledonol synthase; AltName: Full=...	Armillaria ostoyae	567	567	65%	4e-164	28.44%	2210	A0A284RE13.1
RecName: Full=Polyketide synthase 2 [Metarhizium brunneum ARSEF 3297]	Metarhizium bru...	562	562	65%	1e-162	28.31%	2157	A0A0B4H8J1.1
RecName: Full=Polyketide synthase 1; AltName: Full=Conidial pigment biosynthesis polyketide synthase [Metar...	Metarhizium bru...	561	561	55%	2e-162	29.21%	2148	A0A0B4ESU9.1
RecName: Full=Polyketide synthase 1; AltName: Full=Conidial pigment biosynthesis polyketide synthase [Metar...	Metarhizium albu...	561	561	55%	2e-162	30.29%	2162	A0A2U8NET4.1
RecName: Full=Orsellinic acid synthase armB; Short=OAS; AltName: Full=Melledonol synthase; AltName: Full=...	Armillaria mellea	560	560	66%	1e-161	27.81%	2209	I3ZNU9.1
RecName: Full=Polyketide synthase 1; AltName: Full=Conidial pigment biosynthesis polyketide synthase [Metar...	Metarhizium hyb...	556	556	55%	1e-160	29.03%	2148	A0A0B4FTU6.1
RecName: Full=Polyketide synthase 2 [Metarhizium robertsii ARSEF 23]	Metarhizium rob...	556	556	67%	1e-160	27.34%	2157	E9ET39.2
RecName: Full=Conidial yellow pigment biosynthesis polyketide synthase; Short=PKS [Aspergillus nidulans FG...	Aspergillus nidul...	553	553	68%	6e-160	27.35%	2157	Q03149.2
RecName: Full=Non-reducing polyketide synthase albA; Short=NR-PKS albA [Aspergillus brasiliensis CBS 101...	Aspergillus brasil...	553	553	67%	1e-159	27.11%	2147	A0A1L9UCG2.1
RecName: Full=Polyketide synthase 1; AltName: Full=Conidial pigment biosynthesis polyketide synthase [Metar...	Metarhizium rob...	550	550	55%	9e-159	28.93%	2148	E9F646.2
RecName: Full=Conidial yellow pigment biosynthesis polyketide synthase melA; Short=PKS melA [Penicillium e...	Penicillium expa...	549	549	68%	1e-158	26.38%	2138	A0A0A2KT65.1
RecName: Full=Polyketide synthase 1; AltName: Full=Conidial pigment biosynthesis polyketide synthase [Metar...	Metarhizium guiz...	549	549	55%	2e-158	29.01%	2148	A0A0B4G9F9.1
RecName: Full=Polyketide synthase 1; AltName: Full=Conidial pigment biosynthesis polyketide synthase [Metar...	Metarhizium maj...	548	548	55%	5e-158	28.74%	2149	A0A0B4H4F0.1
RecName: Full=Non-reducing polyketide synthase albA; Short=NR-PKS albA [Aspergillus niger ATCC 1015]	Aspergillus niger ...	545	545	68%	3e-157	26.85%	2128	G3XLL5.1
RecName: Full=Non-reducing polyketide synthase albA; Short=NR-PKS albA [Aspergillus niger CBS 513.88]	Aspergillus niger...	544	544	68%	9e-157	26.98%	2153	A2QUI2.1
RecName: Full=Non-reducing polyketide synthase PvBS090_009107; AltName: Full=Pigment biosynthesis clus...	Paecilomyces va...	540	540	66%	1e-155	26.98%	2149	P9WET9.1
RecName: Full=Non-reducing polyketide synthase vrtA; AltName: Full=Viridicatumtoxin synthesis protein A [Pen...	Penicillium aethi...	520	520	47%	2e-150	30.08%	1824	D7PHZ2.1
RecName: Full=Non-reducing polyketide synthase crz7; Short=NR-PKS crz7; AltName: Full=Cristazarin biosynt...	Cladonia metaco...	523	523	62%	2e-150	27.29%	2098	A0A6H0YV38.1
RecName: Full=Non-reducing polyketide synthase afvB; Short=NRPKS; AltName: Full=Aflavarin synthesis prot...	Aspergillus flavu...	511	511	51%	5e-148	29.76%	1751	B8N8R0.1
RecName: Full=Bikaverin polyketide synthase bik1; AltName: Full=Bikaverin biosynthesis protein 1 [Fusarium f...	Fusarium fujikur...	514	514	64%	1e-147	27.46%	2036	S0DZM7.1
RecName: Full=Non-reducing polyketide synthase nscA; AltName: Full=Conidial yellow pigment biosynthesis p...	Aspergillus fisch...	510	510	63%	2e-147	26.49%	1794	A1D8I9.1
RecName: Full=Non-reducing polyketide synthase ustP; Short=NR-PKS ustB; AltName: Full=Ustilaginoidins bio...	Ustilaginoidea vir...	514	514	65%	2e-147	27.09%	2094	A0A1B5L6A0.1
RecName: Full=Kotanin synthase; AltName: Full=Kotanin biosynthesis cluster protein S; AltName: Full=Non-red...	Aspergillus niger...	505	505	52%	1e-146	28.76%	1663	A2QK66.1
RecName: Full=Non-reducing polyketide synthase ptaA; AltName: Full=Pestheic acid biosynthesis cluster protei...	Pestalotiopsis fic...	505	505	55%	4e-146	29.12%	1746	A0A067XNI2.1
RecName: Full=Non-reducing polyketide synthase Preu8; Short=NR-PKS Preu8 [Preussia isomera]	Preussia isomera	510	510	57%	8e-146	27.89%	2158	P9WET3.1
RecName: Full=Non-reducing polyketide synthase pgmA; Short=NR-PKS pgmA; AltName: Full=Pigmented nap...	Aspergillus terre...	510	510	65%	3e-145	26.17%	2298	A0A1W7M1U5.
RecName: Full=Non-reducing polyketide synthase gsfA; AltName: Full=Griseofulvin synthesis protein A; AltNam...	Penicillium aethi...	503	503	63%	6e-145	26.28%	1790	D7PI15.1
RecName: Full=Non-reducing polyketide synthase PKS19; Short=NR-PKS PKS19; AltName: Full=Alternariol sy...	Parastagonospor...	498	498	63%	2e-143	26.88%	1763	A0A0H4ADX3.1
RecName: Full=Non-reducing polyketide synthase PKS12; AltName: Full=Aurofusarin biosynthesis cluster prote...	Fusarium gramin...	501	501	52%	4e-143	28.98%	2067	I1RF58.1
RecName: Full=Conidial pigment polyketide synthase PfmaE; AltName: Full=Conidial pigment biosynthesis clus...	Pestalotiopsis fic...	500	500	57%	2e-142	27.35%	2155	W3X7U2.1
RecName: Full=Non-reducing polyketide synthase ZEA1; AltName: Full=Macrolactone megasynthetase; AltNa...	Fusarium gramin...	497	497	53%	5e-142	28.40%	2038	A0A098D6U0.1
RecName: Full=Atrochrysone carboxylic acid synthase; Short=ACAS; AltName: Full=Cladofulvin biosynthesis cl...	Fulvia fulva	493	493	51%	8e-142	28.35%	1790	P0CU67.1
RecName: Full=Atrochrysone carboxylic acid synthase; Short=ACAS; AltName: Full=Non-reducing polyketide s...	Aspergillus chev...	491	491	56%	4e-141	28.02%	1775	A0A7R7ZK98.1
RecName: Full=Non-reducing polyketide synthase 1; AltName: Full=Elsinochromes biosynthesis cluster protein ...	Elsinoe fawcettii	494	494	69%	1e-140	25.38%	2192	A7UMW1.1
RecName: Full=Non-reducing polyketide synthase rdc1; Short=NR-PKS rdc1; AltName: Full=Radicicol biosynth...	Pochonia chlamy...	492	492	54%	3e-140	28.39%	2090	B3FWT6.1
RecName: Full=Atrochrysone carboxylic acid synthase; Short=ACAS; AltName: Full=Endocrocin synthesis prot...	Aspergillus fumig...	488	488	52%	5e-140	27.99%	1775	Q4W944.1

Figure S5. Protein sequence alignment of TalsA with other homologs using Blastp (protein-protein BLAST)

Description	Scientific Name	Max Score	Total Score	Query Cover	E value	Per. Ident	Acc. Len	Accession
RecName: Full=Cytochrome P450 monooxygenase efuB; AltName: Full=Enfumafungin biosynthesis cluster pr...	Hormonema car...	336	336	96%	7e-109	35.57%	505	A0A2Z4HPZ7.1
RecName: Full=Averantin hydroxylase; AltName: Full=Cytochrome P450 monooxygenase avnA; AltName: Full...	Dothistroma sep...	325	325	95%	3e-104	37.98%	525	M2YJD1.1
RecName: Full=Cytochrome P450 monooxygenase radP; AltName: Full=Radicicol biosynthesis cluster protein ...	Floropilus chiversii	315	315	93%	6e-101	36.83%	501	C5H886.1
RecName: Full=Cytochrome P450 monooxygenase lcsI; AltName: Full=Leucinostatins biosynthesis cluster pro...	Purpureocillium l...	315	315	96%	2e-100	34.93%	516	A0A179H0I7.1
RecName: Full=Cytochrome P450 monooxygenase hmp1; AltName: Full=Hypothemycin biosynthesis cluster p...	Hypomyces subi...	309	309	95%	1e-98	36.69%	502	B3FWR7.1
RecName: Full=Cytochrome P450 monooxygenase atr2; AltName: Full=Atranorin biosynthesis cluster protein ...	Stereocaulon alp...	305	305	95%	9e-97	36.20%	506	A0A8F4SN83.1
RecName: Full=Cytochrome P450 monooxygenase alt3; AltName: Full=Alternapyrone biosynthesis cluster prot...	Alternaria solani	300	300	94%	5e-95	37.30%	509	Q5KTN1.1
RecName: Full=Cytochrome P450 monooxygenase astA; AltName: Full=Asperterpenoid biosynthesis cluster p...	Talaromyces wor...	296	296	95%	1e-93	34.56%	493	A0A3Q9FEJ4.1
RecName: Full=Cytochrome P450 monooxygenase stcF; AltName: Full=Cytochrome P450 60A2; AltName: Ful...	Aspergillus nidul...	294	294	93%	8e-93	35.61%	483	Q12609.3
RecName: Full=Cytochrome P450 monooxygenase lnbC; AltName: Full=Lnb diastereomeric piperazines biosy...	Aspergillus flavu...	290	290	96%	3e-91	34.26%	503	B8NWW3.1
RecName: Full=Cytochrome P450 monooxygenase rdc4; AltName: Full=Hypothemycin biosynthesis cluster pr...	Pochonia chlam...	288	288	94%	2e-90	35.51%	492	B3FWT9.1
RecName: Full=Cytochrome P450 monooxygenase tpeC; AltName: Full=Polyesters biosynthesis cluster protei...	Talaromyces stip...	286	286	96%	2e-89	35.56%	507	B8MV61.1
RecName: Full=Cytochrome P450 monooxygenase astD; AltName: Full=Astellolide biosynthesis cluster protei...	Aspergillus oryz...	285	285	98%	4e-89	35.09%	512	Q2UEK5.1
RecName: Full=Cytochrome P450 monooxygenase aclL; AltName: Full=Aspirochlorine biosynthesis protein L [...	Aspergillus oryz...	284	284	94%	6e-89	33.67%	490	Q2UPC4.1
RecName: Full=Versicolorin B desaturase; AltName: Full=Cytochrome P450 monooxygenase verB; AltName: ...	Dothistroma sep...	284	284	93%	1e-88	35.17%	520	M2XHZ0.1
RecName: Full=Cytochrome P450 monooxygenase cpaH; AltName: Full=Cyclopiazonic acid biosynthesis clust...	Aspergillus oryzae	277	277	94%	4e-86	34.57%	509	C9K202.1
RecName: Full=Averantin hydroxylase; AltName: Full=Aflatoxin biosynthesis protein G; AltName: Full=Cytochr...	Aspergillus para...	272	272	92%	3e-84	34.94%	495	Q12732.2
RecName: Full=Cytochrome P450 monooxygenase prhB; AltName: Full=Paraherquonin biosynthesis cluster pr...	Penicillium brasil...	271	271	95%	4e-84	32.32%	489	A0A1E1FFM3.1
RecName: Full=Versicolorin B desaturase stcL; AltName: Full=Cytochrome P450 60B; AltName: Full=Cytochro...	Aspergillus nidul...	271	271	97%	9e-84	34.65%	496	Q00707.2
RecName: Full=Cytochrome P450 monooxygenase astJ; AltName: Full=Astellolide biosynthesis cluster protein...	Aspergillus oryz...	266	266	92%	3e-82	31.80%	500	Q2UEL1.1
RecName: Full=Cytochrome P450 monooxygenase FUM2; AltName: Full=Fumonisin biosynthesis cluster prot...	Fusarium verticill...	262	262	92%	7e-81	36.53%	470	W7L9E5.1
RecName: Full=Versicolorin B desaturase; AltName: Full=Aflatoxin biosynthesis protein L [Aspergillus parasitic...	Aspergillus para...	262	262	93%	2e-80	34.98%	500	Q9UW95.1
RecName: Full=Cytochrome P450 monooxygenase vrtK; AltName: Full=Viridicatumtoxin synthesis protein K [P...	Penicillium aethi...	263	263	97%	2e-80	33.27%	534	D7PHY9.1
RecName: Full=Cytochrome P450 monooxygenase traB; AltName: Full=Terrestric acid biosynthesis cluster pro...	Penicillium crust...	262	262	94%	2e-80	33.74%	509	A0A481WNM6.1
RecName: Full=Cytochrome P450 monooxygenase sthF; AltName: Full=Stemphyloxin II biosynthesis cluster p...	Parastagonospo...	262	262	95%	4e-80	33.13%	513	Q0UK49.1
RecName: Full=Cytochrome P450 monooxygenase BOA3; AltName: Full=Botcinic acid biosynthesis cluster A ...	Botrytis cinerea ...	260	260	94%	1e-79	33.67%	506	B1GVX3.1
RecName: Full=Cytochrome P450 monooxygenase ORF9; AltName: Full=PR-toxin biosynthesis cluster protein...	Penicillium roqu...	256	256	94%	6e-78	31.70%	509	W6Q403.1
RecName: Full=Trichothecene C-4 hydroxylase; AltName: Full=Cytochrome P450 monooxygenase TRI11; AltN...	Trichoderma aru...	254	254	93%	1e-77	34.02%	499	G0KYA9.1
RecName: Full=Cytochrome P450 monooxygenase bet2; AltName: Full=Betaenone biosynthesis cluster protei...	Neocamarospori...	254	254	92%	3e-77	33.13%	521	A0A0C6DUU3.1
RecName: Full=Cytochrome P450 monooxygenase AMT3; AltName: Full=AM-toxin biosynthesis protein 3 [Alte...	Alternaria alternata	251	251	93%	3e-76	31.39%	489	A7VMU4.2
RecName: Full=Cytochrome P450 monooxygenase dtxS2; AltName: Full=Destruxin synthesis protein 1 [Metar...	Metarhizium rob...	251	251	94%	4e-76	32.65%	516	E9FCP5.2
RecName: Full=Cytochrome P450 monooxygenase 1; AltName: Full=Aphidicolin biosynthesis protein PbP450-...	Neocamarospori...	249	249	94%	2e-75	31.17%	486	Q6F5E4.1
RecName: Full=Cytochrome P450 monooxygenase aba2; AltName: Full=Abscisic acid biosynthesis cluster pro...	Botrytis cinerea ...	247	247	90%	2e-74	32.00%	527	A0A384JQH2.1
RecName: Full=Cytochrome P450 monooxygenase olcB; AltName: Full=15-deoxyoxalicine B biosynthesis clus...	Penicillium cane...	243	243	93%	4e-73	33.13%	491	P9WEQ1.1
RecName: Full=Trichothecene C-15 hydroxylase; AltName: Full=Core trichothecene cluster (CTC) protein 11; ...	Fusarium sporot...	239	239	94%	1e-71	31.78%	492	O13317.1
RecName: Full=Cytochrome P450 monooxygenase alt1; AltName: Full=Alternapyrone biosynthesis cluster prot...	Alternaria solani	233	233	94%	6e-69	30.55%	524	Q5KTN3.1
RecName: Full=Cytochrome P450 monooxygenase hepD; AltName: Full=Heptelidic acid biosynthesis cluster p...	Aspergillus oryz...	229	229	95%	9e-68	31.85%	500	Q2U0K0.1
RecName: Full=Cytochrome P450 monooxygenase BOT4; AltName: Full=Botrydial biosynthesis cluster protein...	Botrytis cinerea	226	226	92%	2e-66	31.95%	526	B2RML6.1
RecName: Full=Fusicoccadiene 8-ol C-16-hydroxylase; AltName: Full=Cytochrome P450 monooxygenase Pa...	Diaporthe amyg...	225	225	97%	6e-66	30.59%	526	L0MXJ1.1
RecName: Full=Fusicoccadiene 8-ol C-16 hydroxylase; AltName: Full=Brassicicene C biosynthetic gene cluste...	Alternaria brassi...	219	219	94%	4e-64	30.44%	495	D1MX88.1
RecName: Full=Cytochrome P450 monooxygenase ABA2; AltName: Full=Abscisic acid biosynthesis protein 2 [...	Pyricularia oryza...	220	220	90%	4e-64	30.72%	521	G4N2X2.1
RecName: Full=Cytochrome P450 monooxygenase btcC; AltName: Full=Betaestacins biosynthesis cluster prot...	Colletotrichum o...	219	219	93%	9e-64	31.10%	520	A0A484FVL2.1
RecName: Full=Cytochrome P450 monooxygenase abl2; AltName: Full=Abscisic acid biosynthesis cluster prot...	Plenodomus ling...	217	217	95%	8e-63	31.40%	555	E5A7D8.1
RecName: Full=Cytochrome P450 monooxygenase aneG; AltName: Full=Aculenes biosynthesis cluster protei...	Aspergillus acul...	216	216	94%	1e-62	30.36%	530	A0A1L9WUV2.1
RecName: Full=Cytochrome P450 monooxygenase ORF11; AltName: Full=PR-toxin biosynthesis cluster protei...	Penicillium roqu...	214	214	92%	3e-62	30.48%	480	W6Q4S4.1
RecName: Full=Cytochrome P450 monooxygenase sdnF; AltName: Full=Sordarin/hypoxysordarin biosynthesis...	Sordaria araneosa	214	214	94%	7e-62	32.68%	521	A0A1B4XBH1.1
RecName: Full=Cytochrome P450 monooxygenase BipB; AltName: Full=Minimal biosynthetic bip cluster protei...	Bipolaris sorokin...	206	206	93%	3e-59	29.71%	508	A0A8H5Z7T4.1
RecName: Full=Cytochrome P450 monooxygenase btcC; AltName: Full=Betaestacins biosynthesis cluster prot...	Neocamarospori...	200	200	92%	1e-56	30.93%	510	A0A2Z6FZ20.1
RecName: Full=Cytochrome P450 monooxygenase TwmD; AltName: Full=Wortmanamides biosynthesis cluste...	Talaromyces wor...	194	194	92%	2e-54	30.02%	512	A0A2L0P0J8.1
RecName: Full=Cytochrome P450 monooxygenase poxC; AltName: Full=Oxaleimides biosynthesis cluster prot...	Penicillium oxali...	186	186	97%	9e-52	29.33%	477	A0A1W5T1U4.1
RecName: Full=Cytochrome P450 monooxygenase btcB; AltName: Full=Betaestacins biosynthesis cluster prot...	Neocamarospori...	186	186	90%	3e-51	28.80%	528	A0A2Z6FZ19.1
RecName: Full=Cytochrome P450 monooxygenase ATR14; AltName: Full=Core atranone cluster (CAC) protei...	Stachybotrys chl...	171	171	85%	2e-46	31.64%	446	A0A084R1J2.1
RecName: Full=Cytochrome P450 monooxygenase tpcB; AltName: Full=Terpestacin biosynthesis cluster prote...	Bipolaris maydis...	171	171	93%	5e-46	27.13%	506	M2V0Z8.1
RecName: Full=Cytochrome P450 monooxygenase FUP2; AltName: Full=Fusaproliferin biosynthesis cluster pr...	Fusarium prolifer...	168	168	92%	7e-45	29.55%	509	A0A1L7VEQ8.1
RecName: Full=Cytochrome P450 monooxygenase hepC; AltName: Full=Heptelidic acid biosynthesis cluster p...	Aspergillus oryz...	166	166	95%	3e-44	27.37%	509	Q2U0K1.2
RecName: Full=Cytochrome P450 monooxygenase atnE; AltName: Full=Aspercryptin biosynthesis cluster prot...	Aspergillus nidul...	165	165	89%	6e-44	26.64%	518	Q5AUZ9.1
RecName: Full=Cytochrome P450 monooxygenase notG; AltName: Full=Notoamide biosynthesis cluster protei...	Aspergillus sp. ...	165	165	89%	9e-44	28.51%	543	E1ACQ2.1
RecName: Full=Tryprostatin B 6-hydroxylase; AltName: Full=Fumitremorgin biosynthesis protein C [Aspergillus...	Aspergillus fisch...	164	164	91%	3e-43	28.07%	548	A1DA60.1
RecName: Full=Tryprostatin B 6-hydroxylase; AltName: Full=Cytochrome P450 monooxygenase ftmP450-1; Al...	Aspergillus fumi...	163	163	93%	7e-43	27.14%	559	B9WZX1.1
RecName: Full=Cytochrome P450 monooxygenase tazI; AltName: Full=Azaphilone biosynthesis cluster protei...	Aspergillus terre...	161	161	91%	1e-42	24.00%	489	Q0CS90.2
RecName: Full=Cytochrome P450 monooxygenase poxM; AltName: Full=Oxaleimides biosynthesis cluster pro...	Penicillium oxali...	160	160	89%	3e-42	27.14%	512	A0A1W5T1Y6.1
RecName: Full=Sesterfisheric acid synthase; AltName: Full=Cytochrome P450 monooxygenase NfP450; AltNa...	Aspergillus fisch...	161	161	88%	4e-42	27.54%	547	A1DN29.2
RecName: Full=Cytochrome P450 monooxygenase aneF; AltName: Full=Aculenes biosynthesis cluster protein...	Aspergillus acul...	157	157	93%	4e-41	26.04%	495	A0A1L9WVI3.1
RecName: Full=Cytochrome P450 monooxygenase notG'; AltName: Full=Notoamide biosynthesis cluster prote...	Aspergillus versi...	152	152	89%	3e-39	27.80%	544	L7WME9.1
RecName: Full=Cytochrome P450 monooxygenase BOT1; AltName: Full=Botrydial biosynthesis cluster protein...	Botrytis cinerea	145	145	95%	7e-37	26.34%	510	Q6WP49.1
RecName: Full=Cytochrome P450 monooxygenase dpfgJ; AltName: Full=Diterpenoid pyrone biosynthesis clus...	Fusarium gramin...	145	145	90%	9e-37	26.77%	481	I1RL13.1
RecName: Full=Cytochrome P450 monooxygenase orf2; AltName: Full=Pestalamide A biosynthesis cluster pro...	Aspergillus niger...	145	145	95%	1e-36	26.16%	488	A2QTE8.1
RecName: Full=Cytochrome P450 monooxygenase ABA2; AltName: Full=Abscisic acid biosynthesis protein 2 [...	Pyricularia oryza...	143	143	68%	2e-36	27.73%	418	L7HT17.1
RecName: Full=Cytochrome P450 monooxygenase AFT11-1; AltName: Full=AF-toxin biosynthesis protein 11-1...	Alternaria alternata	144	144	87%	3e-36	25.38%	509	Q50LG1.1
RecName: Full=Pisatin demethylase; AltName: Full=Cytochrome P450 57A1 [Fusarium vanettenii]	Fusarium vanett...	144	144	92%	3e-36	26.01%	515	Q12645.1
RecName: Full=Pisatin demethylase; AltName: Full=Cytochrome P450 57A2 [Fusarium vanettenii]	Fusarium vanett...	140	140	92%	4e-35	25.00%	506	P38364.1
RecName: Full=Cytochrome P450 monooxygenase cypX; AltName: Full=Dothistromin biosynthesis protein cyp...	Dothistroma sep...	140	140	91%	4e-35	25.21%	511	M2YIZ5.1
RecName: Full=Cytochrome P450 monooxygenase AKT7; AltName: Full=AK-toxin biosynthesis protein 7 [Alter...	Alternaria alternata	140	140	89%	5e-35	24.09%	522	V5XZS8.1
RecName: Full=Cytochrome P450 3A29; AltName: Full=CYPIIIA29 [Sus scrofa]	Sus scrofa	139	139	87%	1e-34	29.49%	503	P79401.1
RecName: Full=Cytochrome P450 3A31; AltName: Full=CYPIIIA31; AltName: Full=Cytochrome P450 SH3A-1 [...	Mesocricetus au...	139	139	89%	1e-34	28.28%	501	O70537.1
RecName: Full=Cytochrome P450 3A28; AltName: Full=CYPIIIA28 [Bos taurus]	Bos taurus	134	134	89%	7e-33	28.27%	507	P79102.1
RecName: Full=Tryptophan 6-hydroxylase fscE; AltName: Full=Cytochrome P450 monooxygenase fscE; AltNa...	Fusarium equiseti	134	134	94%	1e-32	24.30%	510	A0A831A9C9.1
RecName: Full=Cytochrome P450 monooxygenase aflV; AltName: Full=Aflatoxin biosynthesis protein V [Asper...	Aspergillus para...	133	133	88%	1e-32	25.97%	508	Q6UEF4.1
RecName: Full=Cytochrome P450 monooxygenase macH; AltName: Full=Macrophorins biosynthesis cluster pr...	Penicillium terre...	133	133	91%	2e-32	25.83%	510	A0A2P1DPA5.1
RecName: Full=Cytochrome P450 3A12; AltName: Full=CYPIIIA12; AltName: Full=Cytochrome P450-PBD-1 [...	Canis lupus fami...	131	131	80%	6e-32	28.81%	503	P24463.1
RecName: Full=Cytochrome P450 monooxygenase sthD; AltName: Full=Stemphyloxin II biosynthesis cluster p...	Parastagonospo...	131	131	90%	7e-32	25.15%	475	Q0UK51.1
RecName: Full=Cytochrome P450 monooxygenase gsfF; AltName: Full=Griseofulvin synthesis protein F; Flag...	Penicillium aethi...	131	131	90%	9e-32	25.61%	504	D7PI20.2
RecName: Full=Cytochrome P450 3A2; AltName: Full=CYPIIIA2; AltName: Full=Cytochrome P450-PCN2; AltN...	Rattus norvegicus	130	130	74%	2e-31	29.47%	504	P05183.2
RecName: Full=Cytochrome P450 3A24; AltName: Full=CYPIIIA24 [Ovis aries]	Ovis aries	130	130	89%	2e-31	26.00%	503	Q29496.1
RecName: Full=Cytochrome P450 monooxygenase stcB; AltName: Full=Cytochrome P450 62; AltName: Full=...	Aspergillus nidul...	130	130	86%	3e-31	26.26%	517	Q12608.2
RecName: Full=Cytochrome P450 3A18; AltName: Full=CYPIIIA18; AltName: Full=Cytochrome P450(6)beta-2 ...	Rattus norvegicus	129	129	94%	3e-31	26.18%	497	Q64581.1
RecName: Full=Cytochrome P450 monooxygenase BOT3; AltName: Full=Botrydial biosynthesis cluster protein...	Botrytis cinerea	130	130	88%	3e-31	27.06%	567	Q6WP51.1
RecName: Full=Cytochrome P450 monooxygenase orf5; AltName: Full=Brefeldin A biosynthesis cluster protein...	Penicillium brefe...	129	129	90%	4e-31	25.53%	489	A0A068AA78.1
RecName: Full=Cytochrome P450 3A5; AltName: Full=CYPIIIA5; AltName: Full=Cytochrome P450-PCN3 [Ho...	Homo sapiens	129	129	80%	5e-31	28.50%	502	P20815.1
RecName: Full=Lithocholate 6-beta-hydroxylase; Short=6 beta-hydroxylase; AltName: Full=CYPIIIA10; AltNam...	Mesocricetus au...	128	128	85%	7e-31	25.93%	503	Q64148.2
RecName: Full=Cytochrome P450 monooxygenase apf7; AltName: Full=Apicidin F synthesis protein 7 [Fusariu...	Fusarium fujikur...	128	128	89%	9e-31	25.51%	530	S0DPM1.1
RecName: Full=Cytochrome P450 3A56; AltName: Full=CYPIIIA56 [Fundulus heteroclitus]	Fundulus hetero...	127	127	76%	1e-30	26.25%	496	Q8AXY5.1
RecName: Full=Conidiogenone synthase PchP450; AltName: Full=Conidiogenone biosynthesis cluster protein ...	Penicillium rube...	127	127	91%	2e-30	25.00%	491	B6HFX9.1
RecName: Full=Cytochrome P450 3A30; AltName: Full=CYPIIIA30 [Fundulus heteroclitus]	Fundulus hetero...	127	127	76%	2e-30	26.20%	496	Q9PVE8.2
RecName: Full=Cytochrome P450 3A25; AltName: Full=CYPIIIA25 [Mus musculus]	Mus musculus	127	127	94%	2e-30	25.87%	503	O09158.1
RecName: Full=Cytochrome P450 3A27; AltName: Full=CYPIIIA27 [Oncorhynchus mykiss]	Oncorhynchus ...	126	126	76%	6e-30	26.56%	518	O42563.1
RecName: Full=Cytochrome P450 3A21; AltName: Full=CYPIIIA21; AltName: Full=Cytochrome P450 CM3A-1...	Callithrix jacchus	125	125	75%	1e-29	29.90%	503	O18993.1
RecName: Full=Cytochrome P450 3A9; AltName: Full=3AH15; AltName: Full=CYPIIIA9; AltName: Full=Cytoch...	Rattus norvegicus	125	125	90%	1e-29	26.62%	503	P51538.2
RecName: Full=Cytochrome P450 monooxygenase FGSG_15680; AltName: Full=Gramillins biosynthetic clust...	Fusarium gramin...	124	124	74%	1e-29	24.03%	430	A0A098CZ12.1
RecName: Full=Cytochrome P450 monooxygenase phqM; AltName: Full=Paraherquamide biosynthesis cluste...	Penicillium fellut...	124	124	88%	2e-29	25.71%	536	L0E2R0.1

Figure S6. Protein sequence alignment of TalsB with other homologs using Blastp (protein-protein BLAST)

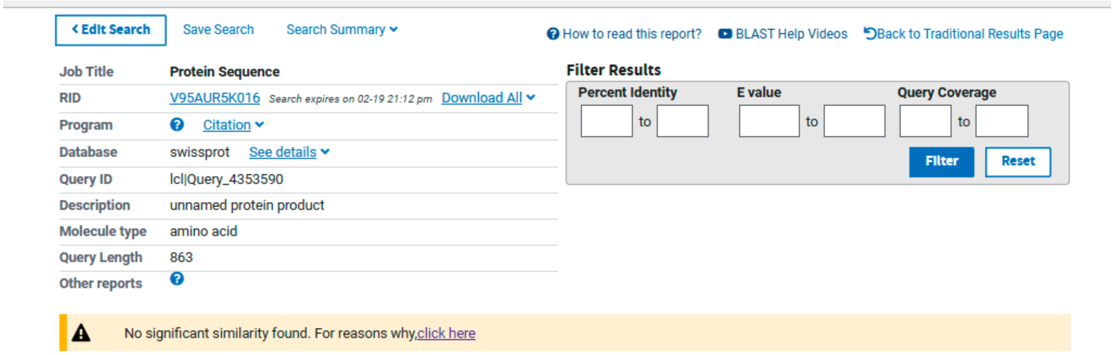

Figure S7. Protein sequence alignment of TalsC with other homologs using Blastp (protein-protein BLAST)

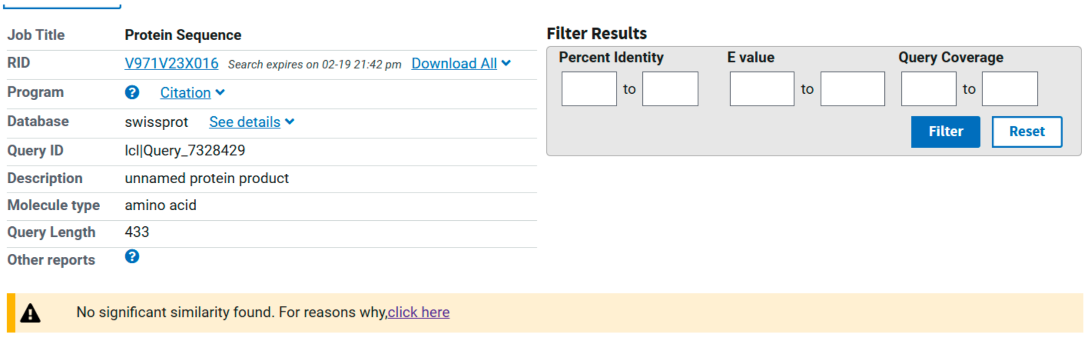

Figure S8. Protein sequence alignment of TalsD with other homologs using Blastp (protein-protein BLAST)

Description	Scientific Name	Max Score	Total Score	Query Cover	E value	Per. Ident	Acc. Len	Accession
RecName: Full=FAD-linked oxidoreductase azaL; AltName: Full=Azaphilone biosynthesis cluster protein azaL; Fl...	Aspergillus niger ...	337	337	95%	7e-110	38.22%	472	G3XMD0.2
RecName: Full=FAD-linked oxidoreductase tazG; AltName: Full=Azaphilone biosynthesis cluster protein G; Flags...	Aspergillus terre...	300	300	97%	1e-95	35.37%	489	Q0CSA1.1
RecName: Full=FAD-linked oxidoreductase azaG; AltName: Full=Azaphilone biosynthesis cluster protein azaG; F...	Aspergillus niger ...	297	297	95%	1e-94	34.93%	468	G3XMC1.1
RecName: Full=FAD-linked oxidoreductase srdI; AltName: Full=Sordarial biosynthesis cluster protein srdI; Flags: ...	Neurospora cras...	295	295	98%	2e-93	32.73%	500	Q7SHH8.1
RecName: Full=FAD-linked oxidoreductase OXR1; AltName: Full=Pyriculol/pyriculariol biosynthesis cluster protei...	Pyricularia oryza...	288	288	89%	2e-90	35.63%	507	G4N285.1
RecName: Full=FAD-linked oxidoreductase tazL; AltName: Full=Azaphilone biosynthesis cluster protein L; Flags: ...	Aspergillus terre...	270	270	92%	2e-83	33.26%	493	Q0CS92.1
RecName: Full=FAD-linked oxidoreductase virF; AltName: Full=Trichoxide biosynthesis protein virF; AltName: Fu...	Trichoderma vire...	242	242	88%	2e-73	34.24%	451	G9N4A7.1
RecName: Full=FAD-linked oxidoreductase fogF; AltName: Full=Flavoglaucin biosynthesis cluster protein F; Flag...	Aspergillus ruber...	237	237	91%	6e-71	32.33%	497	A0A017SGC7.1
RecName: Full=FAD-linked oxidoreductase fmqD; AltName: Full=Fumiquinazoline biosynthesis cluster protein D; ...	Aspergillus fumig...	233	233	97%	3e-69	32.74%	497	Q4WLW6.1
RecName: Full=FAD-linked oxidoreductase anuG; AltName: Full=Annullatin D biosynthesis cluster protein G; Fla...	Penicillium roque...	216	216	96%	4e-63	30.25%	509	W6QEK0.1
RecName: Full=FAD-linked oxidoreductase AFUA_1G00980; AltName: Full=Fumigermin biosynthesis cluster prot...	Aspergillus fumig...	215	215	92%	8e-63	28.23%	496	Q4WKX2.1
RecName: Full=FAD-linked oxidoreductase afoF; AltName: Full=Asperfuranone biosynthesis protein B; Flags: Pr...	Aspergillus nidul...	214	214	92%	2e-62	30.84%	481	Q5BEJ5.1
RecName: Full=FAD-linked oxidoreductase ATEG_07660; AltName: Full=Azasperpyranone A biosynthesis cluste...	Aspergillus terre...	206	206	94%	2e-59	30.56%	481	Q0CF74.1
RecName: Full=FAD-linked oxidoreductase chry5; AltName: Full=Chrysogine biosynthesis cluster protein 5; Flag...	Fusarium gramin...	199	199	97%	9e-57	27.84%	506	I1S3L1.1
RecName: Full=FAD-linked oxidoreductase chyH; AltName: Full=Chrysogine biosynthesis cluster protein H; Flag...	Penicillium ruben...	196	196	94%	1e-55	28.07%	500	B6HLP5.1
RecName: Full=FAD-linked oxidoreductase virI; AltName: Full=Trichoxide biosynthesis protein virI; AltName: Full...	Trichoderma vire...	186	186	96%	9e-52	28.98%	510	G9N4A4.1
RecName: Full=FAD-linked oxidoreductase OXR2; AltName: Full=Pyriculol/pyriculariol biosynthesis cluster protei...	Pyricularia oryza...	183	183	97%	2e-50	29.46%	520	G4N287.1
RecName: Full=FAD-linked oxidoreductase alt4; AltName: Full=Alternapyrone biosynthesis cluster protein 4 [Alte...	Alternaria solani	173	173	93%	4e-47	26.09%	482	Q5KTN0.1
RecName: Full=FAD-linked oxidoreductase iacH; AltName: Full=Iso-A82775C biosynthesis cluster protein HK; Fl...	Pestalotiopsis fici...	170	170	93%	8e-46	28.40%	512	A0A1J0KJK5.1
RecName: Full=Xylooligosaccharide oxidase; Short=XOS; Flags: Precursor [Thermothelomyces thermophilus AT...	Thermothelomyc...	107	107	51%	7e-24	30.42%	497	G2QG48.1
RecName: Full=FAD-linked oxidoreductase pyvE; AltName: Full=Pyranoviolin A biosynthesis cluster protein E [As...	Aspergillus viola...	103	103	92%	1e-22	24.79%	452	A0A2V5HCN7.1
RecName: Full=FAD-linked oxidoreductase pgmH; AltName: Full=Pigmented naphthoquinones biosynthesis clust...	Aspergillus terre...	102	102	90%	3e-22	23.57%	483	Q0CJC3.1
RecName: Full=FAD-linked oxidoreductase pgmH; AltName: Full=Pigmented naphthoquinones biosynthesis clust...	Aspergillus terreus	97.8	97.8	90%	2e-20	24.84%	492	A0A5M3Z5R2.1

Figure S9. Protein sequence alignment of TalsE with other homologs using Blastp (protein-protein BLAST)

Description	Scientific Name	Max Score	Total Score	Query Cover	E value	Per. Ident	Acc. Len	Accession
RecName: Full=Decarboxylase macB; AltName: Full=Macrophorins biosynthesis cluster protein B [Penicillium t…	Penicillium terre…	259	259	98%	6e-84	43.03%	338	A0A2P1DP90.1
RecName: Full=Decarboxylase yanB; AltName: Full=Yanuthone D biosynthesis cluster protein B [Aspergillus ni…	Aspergillus niger…	258	258	98%	6e-83	43.34%	368	G3Y417.1
RecName: Full=6-methylsalicylic acid decarboxylase; AltName: Full=Sesquiterpenyl epoxy-cyclohexenoids clust…	Orbilia oligospor…	252	252	98%	2e-81	42.50%	325	G1XU02.1
RecName: Full=6-methylsalicylic acid decarboxylase; AltName: Full=Patulin biosynthesis cluster protein G [Peni…	Penicillium expa…	231	231	98%	4e-73	38.80%	324	A0A075TXZ1.1
RecName: Full=6-methylsalicylic acid decarboxylase; AltName: Full=Patulin synthesis protein G [Aspergillus cla…	Aspergillus clava…	230	230	99%	7e-73	39.20%	325	A1CFL4.1
RecName: Full=6-methylsalicylic acid decarboxylase acuB; AltName: Full=Aculin biosynthesis cluster protein B …	Aspergillus acule…	224	224	98%	3e-70	39.38%	330	A0A1L9WN60.1
RecName: Full=Gamma-resorcylate decarboxylase; Short=GRA decarboxylase; AltName: Full=2,6-dihydroxybe…	Rhodococcus jo…	87.8	87.8	82%	2e-18	28.42%	329	Q0SFL6.1
RecName: Full=Gamma-resorcylate decarboxylase; AltName: Full=2,6-dihydroxybenzoate decarboxylase; AltN…	Agrobacterium t…	85.5	85.5	82%	1e-17	28.01%	327	Q60FX6.1
RecName: Full=Gamma-resorcylate decarboxylase; Short=GRDC; Short=Gamma-RDC; AltName: Full=2,6-dihy…	Rhizobium sp. M…	84.7	84.7	82%	2e-17	28.01%	327	Q60GU1.1
RecName: Full=2-amino-3-carboxymuconate-6-semialdehyde decarboxylase; AltName: Full=Picolinate carboxyl…	Homo sapiens	78.2	78.2	86%	4e-15	25.33%	336	Q8TDX5.1
RecName: Full=2-amino-3-carboxymuconate-6-semialdehyde decarboxylase; AltName: Full=Picolinate carboxyl…	Rattus norvegicus	77.4	77.4	86%	8e-15	22.67%	336	Q8R5M5.1
RecName: Full=2-amino-3-carboxymuconate-6-semialdehyde decarboxylase; AltName: Full=Picolinate carboxyl…	Mus musculus	75.9	75.9	86%	2e-14	22.67%	336	Q8R519.2
RecName: Full=Gamma-resorcylate decarboxylase; Short=Gamma-RSD; AltName: Full=2,6-dihydroxybenzoate…	Polaromonas sp.…	69.3	69.3	75%	4e-12	25.79%	326	Q12BV1.1
RecName: Full=2-keto-4-carboxy-3-hexenedioate hydratase; Short=KCH hydratase [Sphingobium sp. SYK-6]	Sphingobium sp.…	48.5	48.5	85%	4e-05	24.50%	341	G2IQQ5.1

Figure S10. Protein sequence alignment of TalsF with other homologs using Blastp (protein-protein BLAST)

select all *1 sequences selected* GenPept Graphics Distance tree of results Multiple alignment MSA Viewer

	Description	Scientific Name	Max Score	Total Score	Query Cover	E value	Per. Ident	Acc. Len	Accession
☑	RecName: Full=Putative hetero-Diels-Alderase asR5; AltName: Full=Xenovulene A biosynthesis cluster protein R...	Sarocladium sp. '...	57.4	57.4	83%	5e-08	23.91%	401	A0A2U8U2M1.1

Figure S11. Protein sequence alignment of TalsG with other homologs using Blastp (protein-protein BLAST)

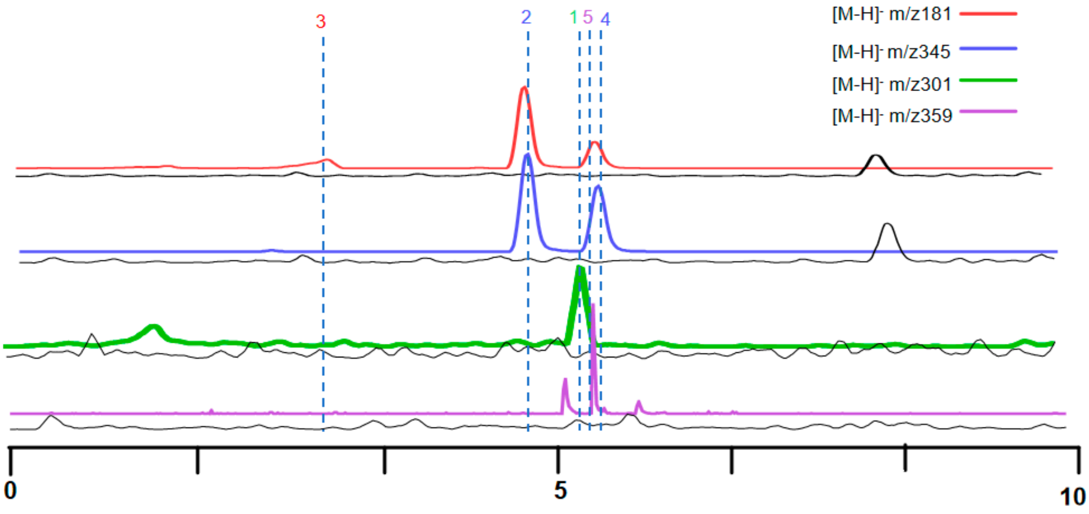

Figure S12. LC-MS spectra of compounds isolated and purified from ethyl acetate extracts obtained from *A. nidulans* transformants against the same molecular weight of the native strain of *A. nidulans*

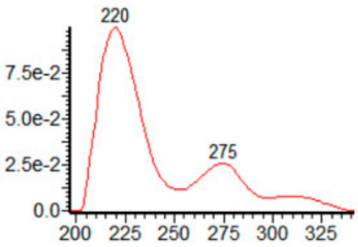

1

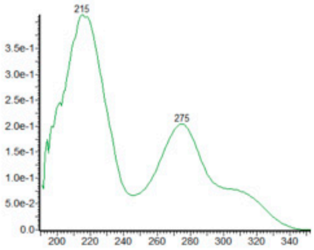

2

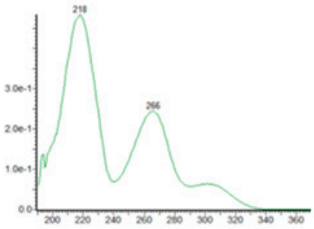

3

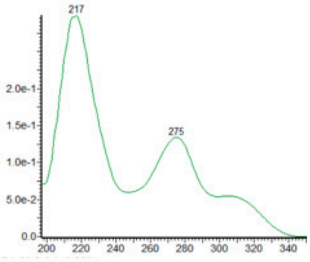

4

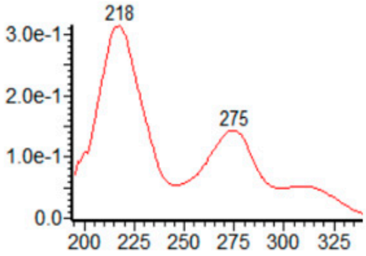

5

Figure S13. UV-Vis spectra of purified compounds. All spectra were measured by an Agilent HPLC system equipped with a variable wavelength detector.

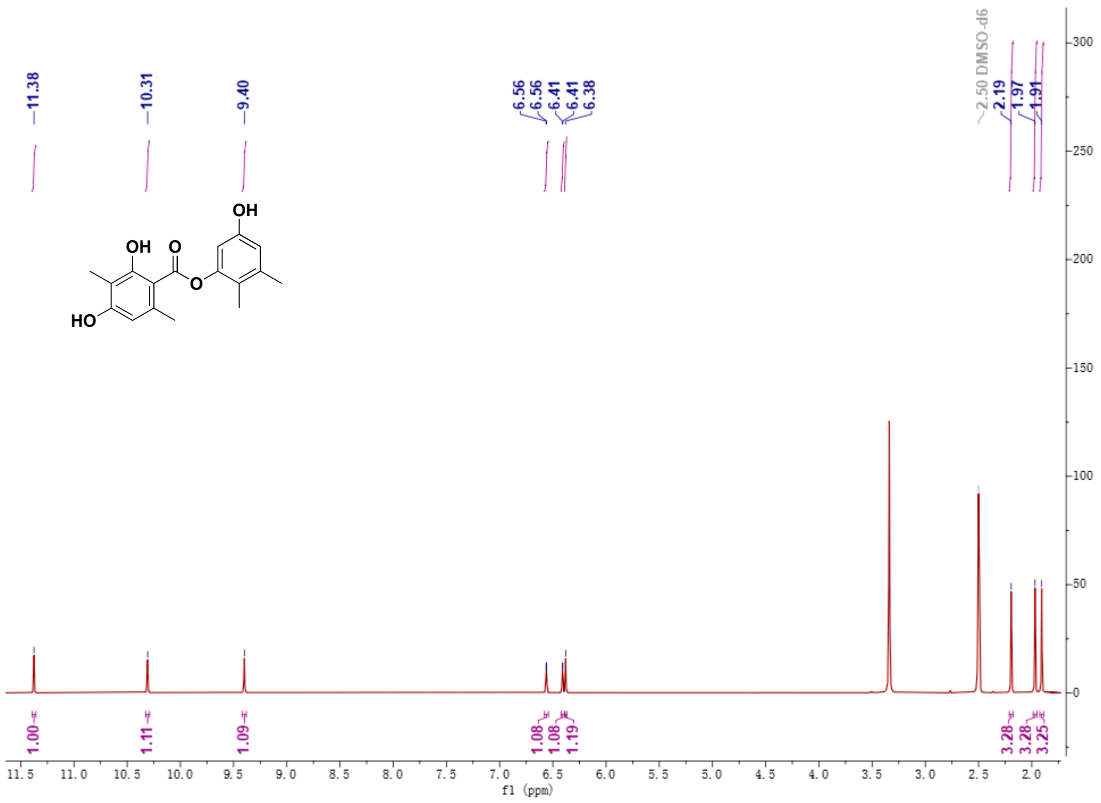

Figure S14. ^{1}H NMR of **1** in *DMSO-d6*

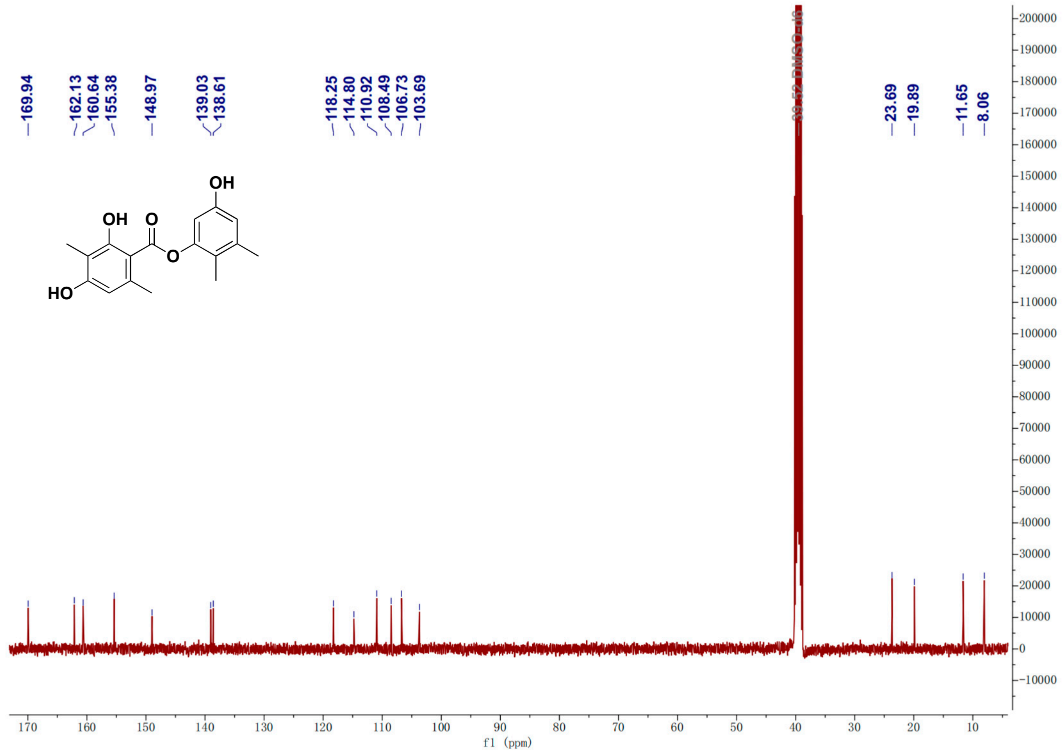

Figure S15. ^{13}C NMR of **1** in *DMSO-d6*

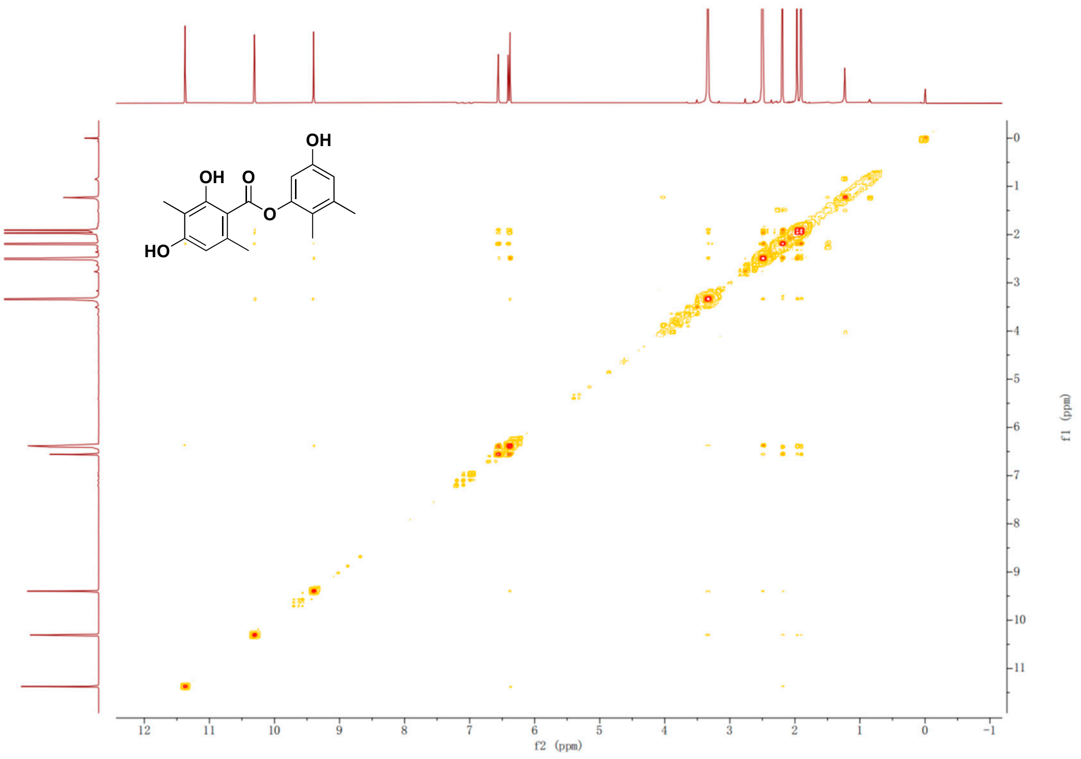

Figure S16. COSY of **1** in DMSO-*d*6.

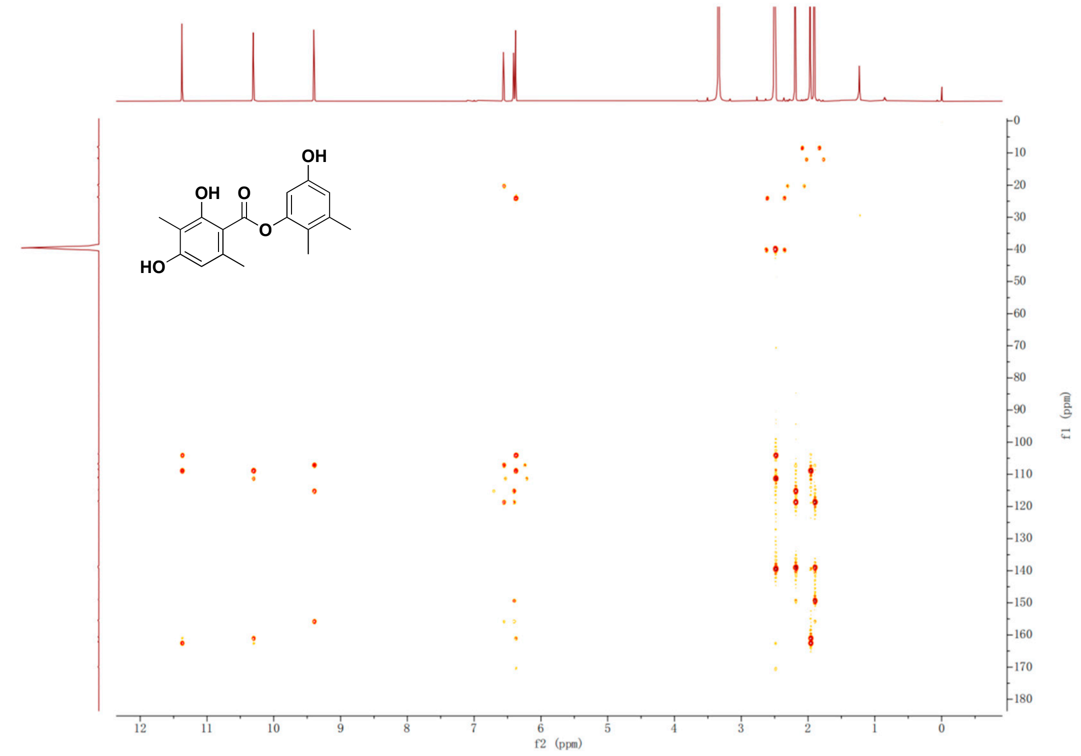

Figure S17. HMBC of **1** in DMSO-*d*6.

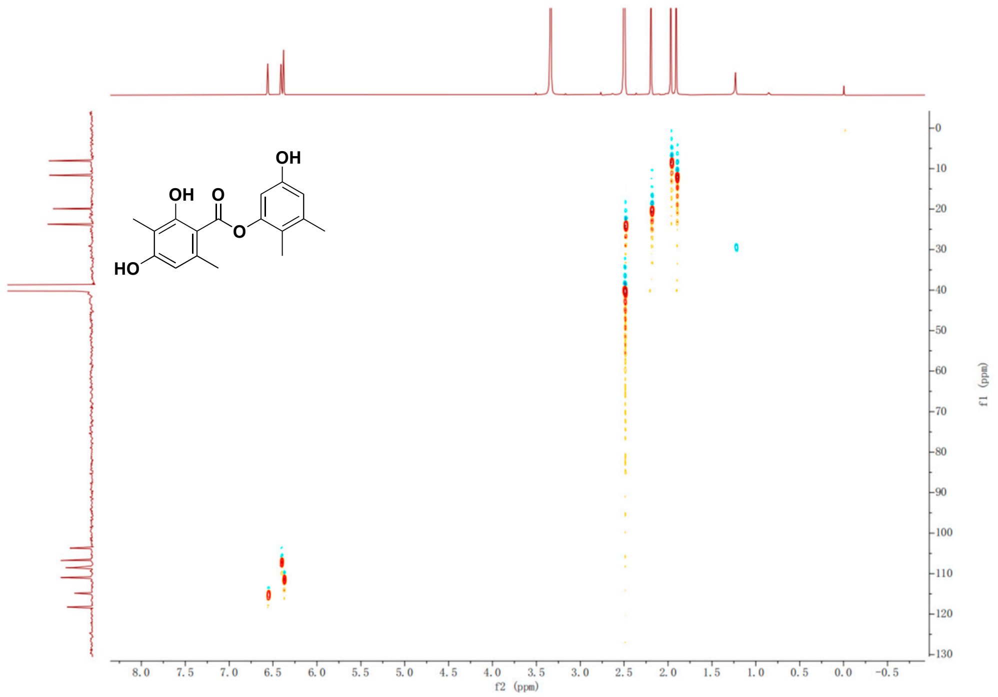

Figure S18. HSQC of **1** in DMSO-*d*6.

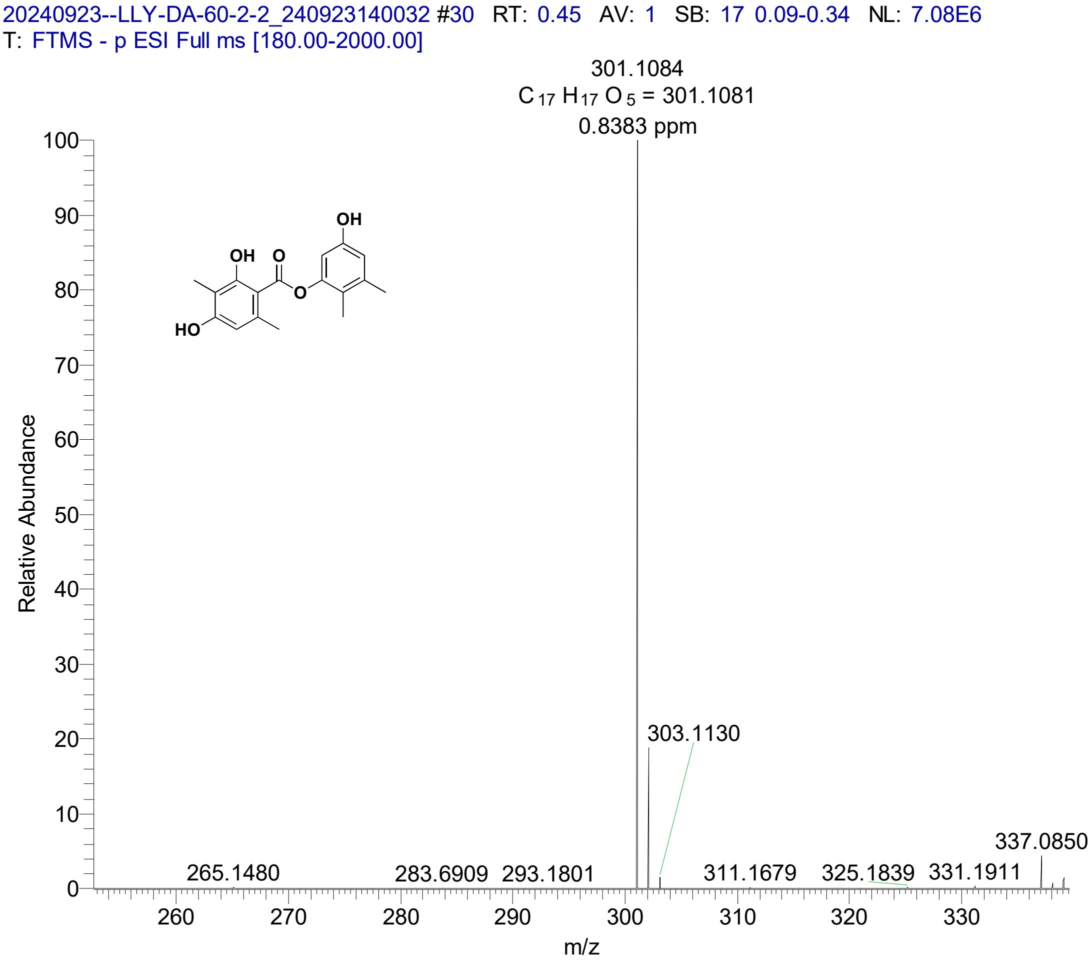

Figure S19. HRESIMS of **1**.

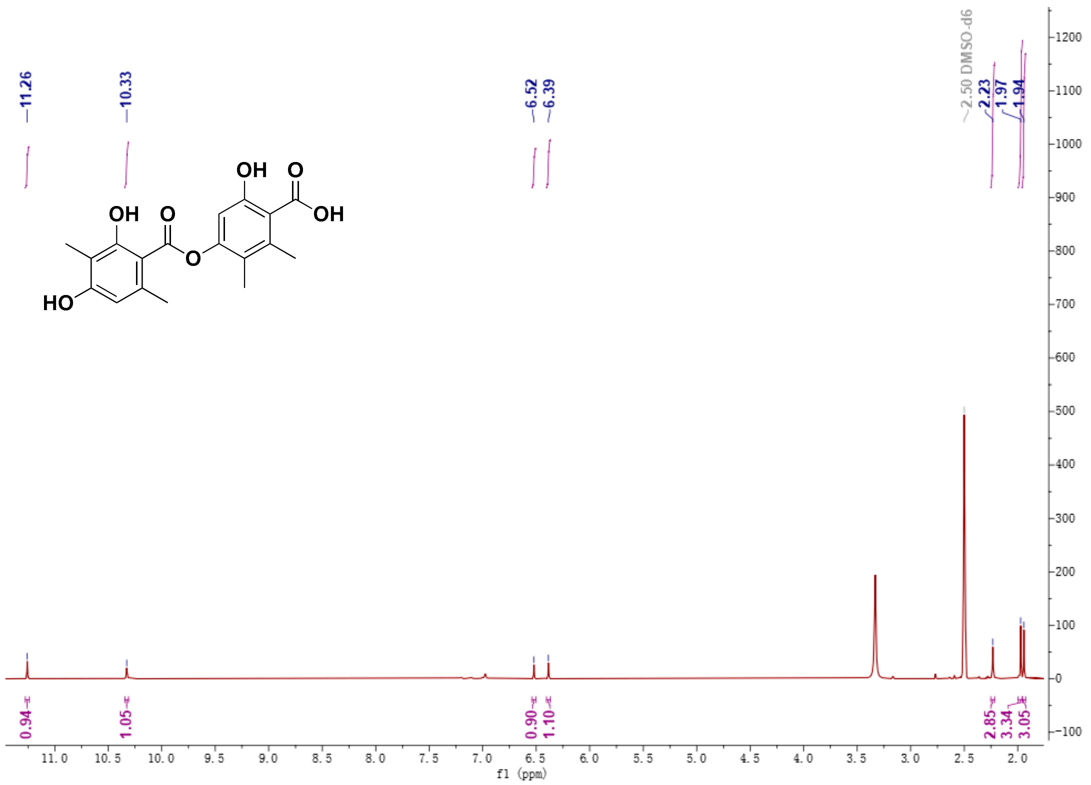

Figure S20. ^{1}H NMR of **2** in DMSO-*d*$_6$

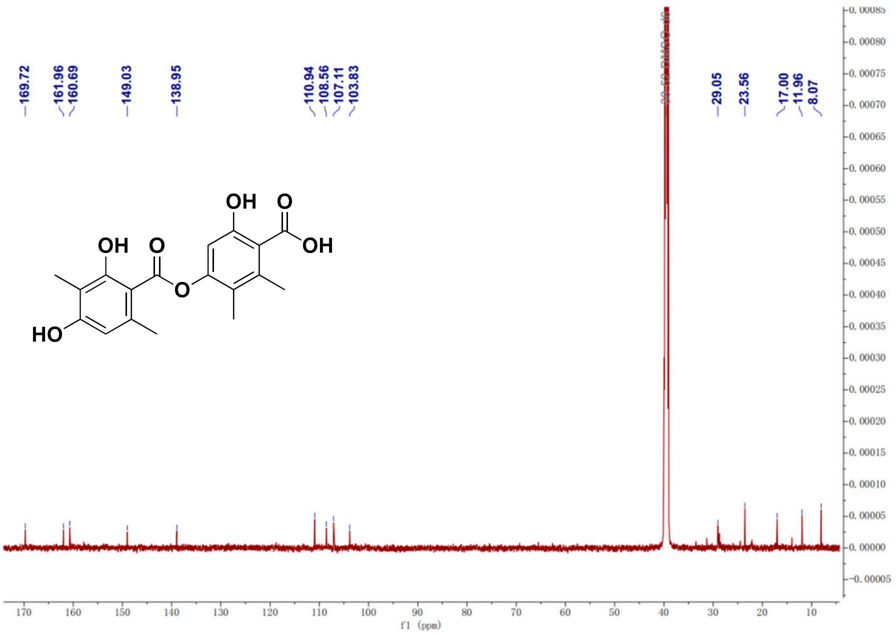

Figure S 21. ^{13}C NMR of **2** in DMSO-*d*$_6$

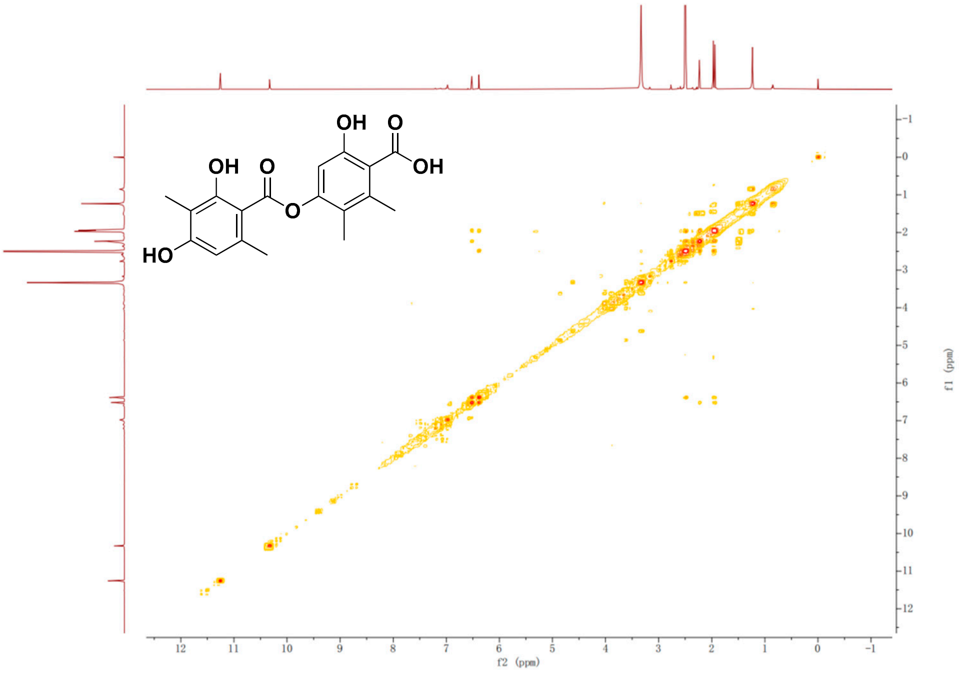

Figure S 22. COSY of **2** in DMSO-d_6.

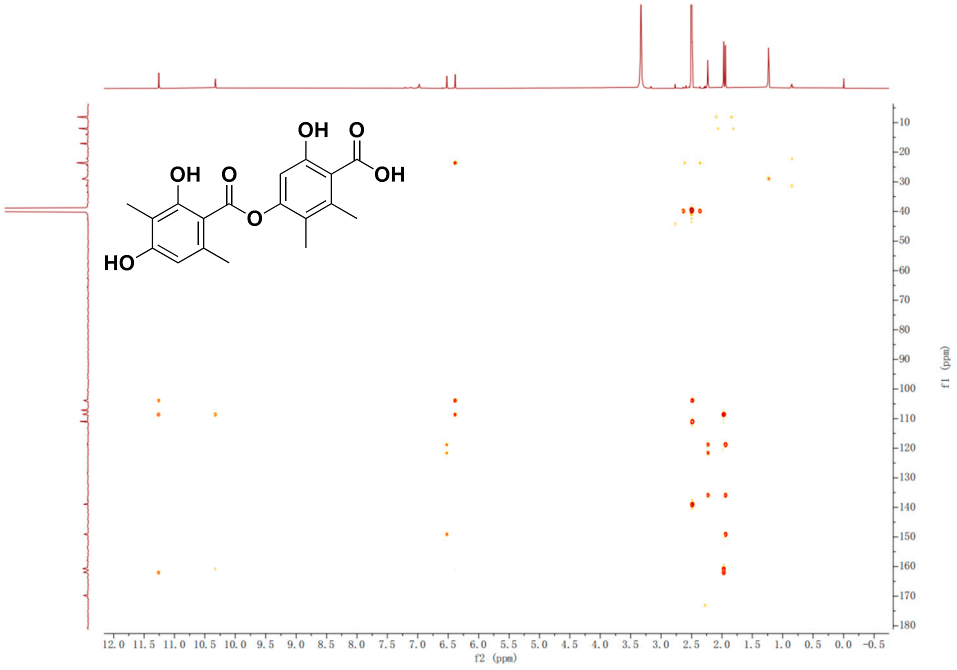

Figure S 23. HMBC of **2** in DMSO-d_6.

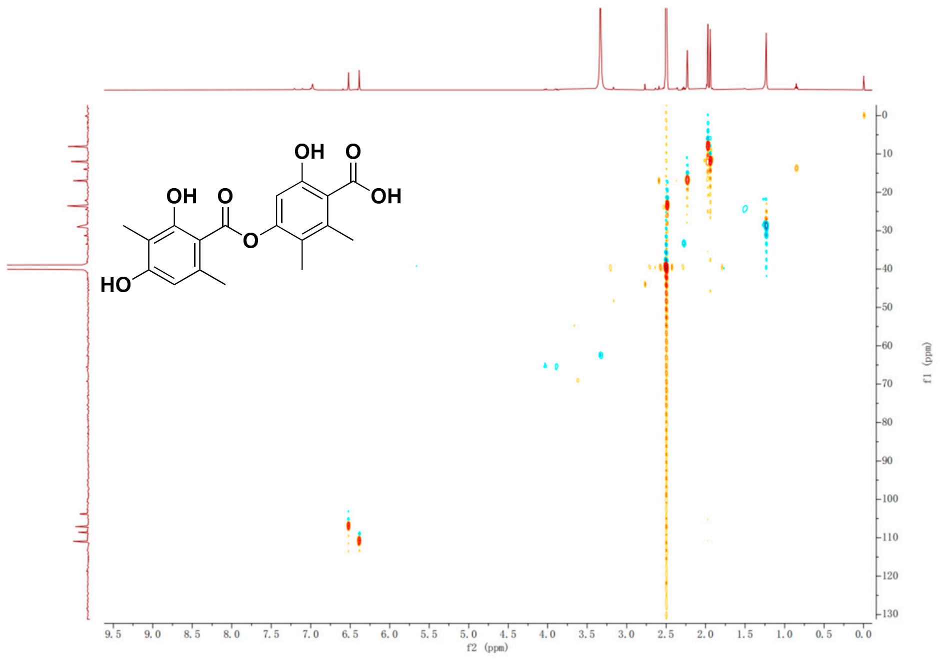

Figure S24. HSQC of **2** in DMSO-d_6.

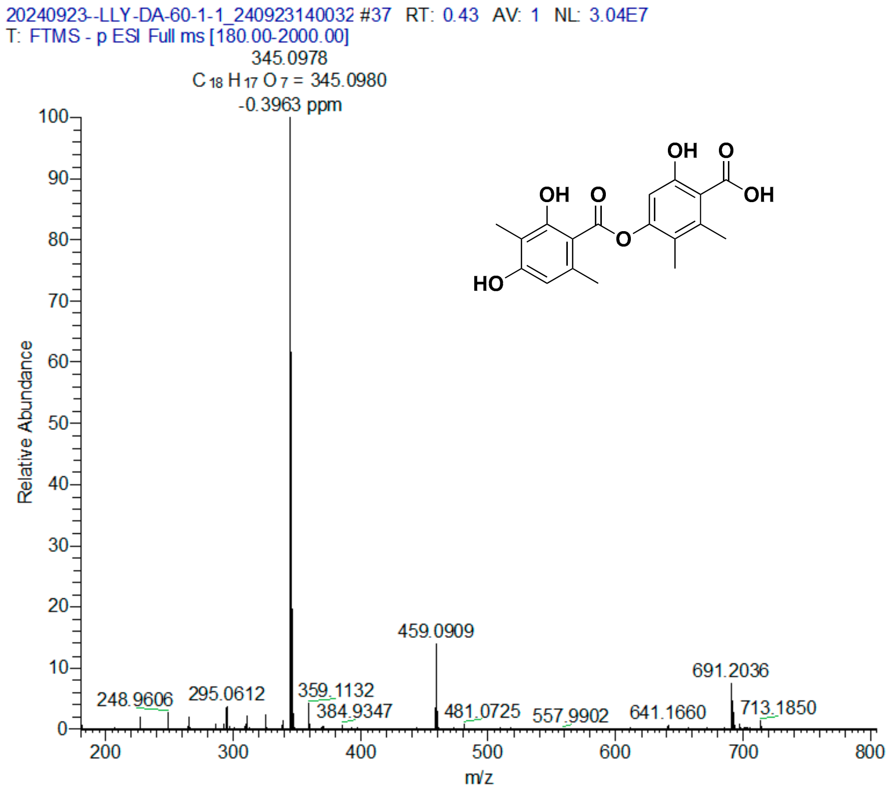

Figure S25. HRESIMS of **2**.

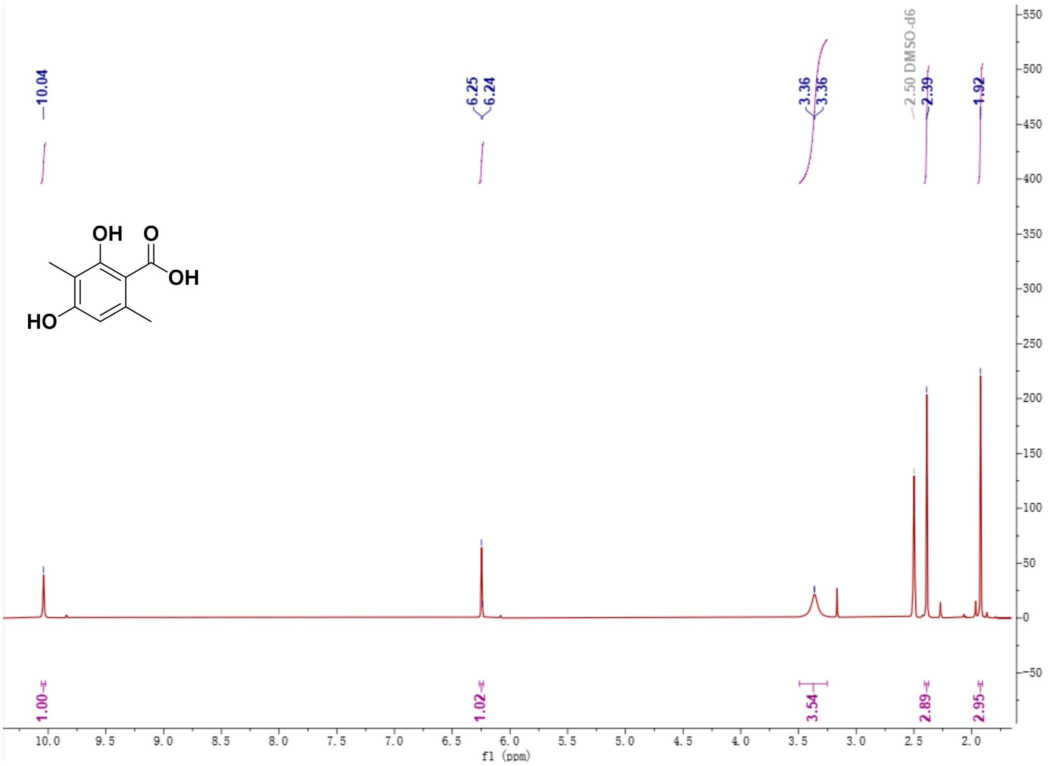

Figure S26. ^{1}H NMR of **3** in DMSO-*d6*

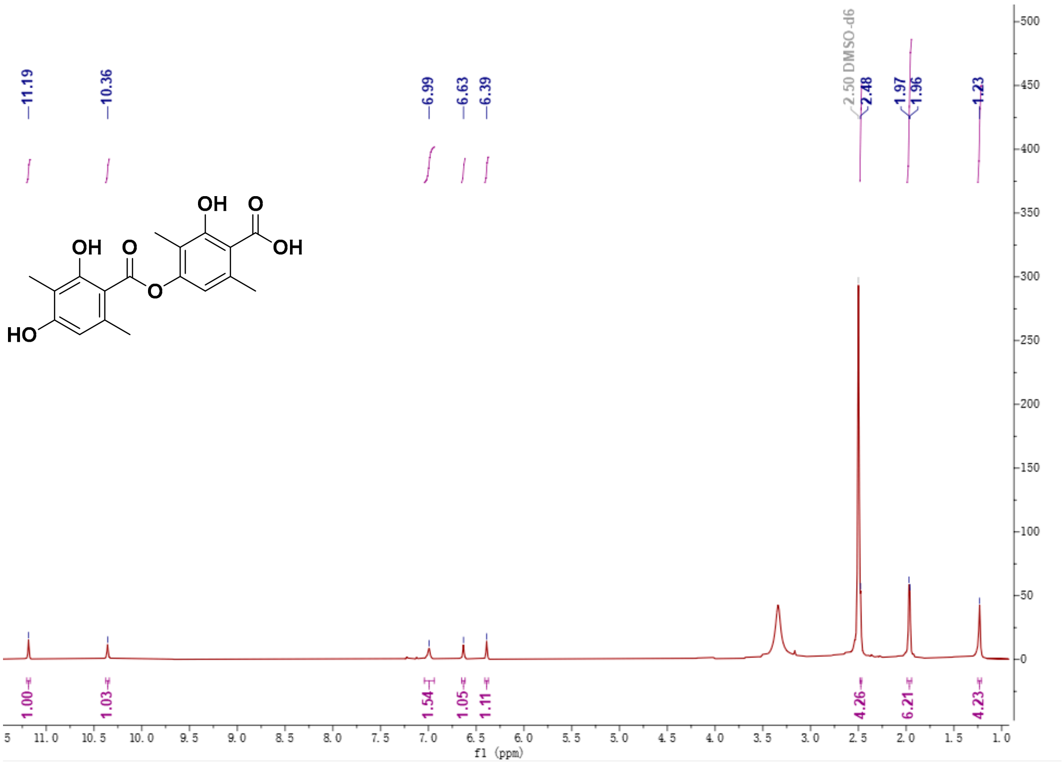

Figure S27 ^{1}H NMR of **4** in DMSO-*d6*

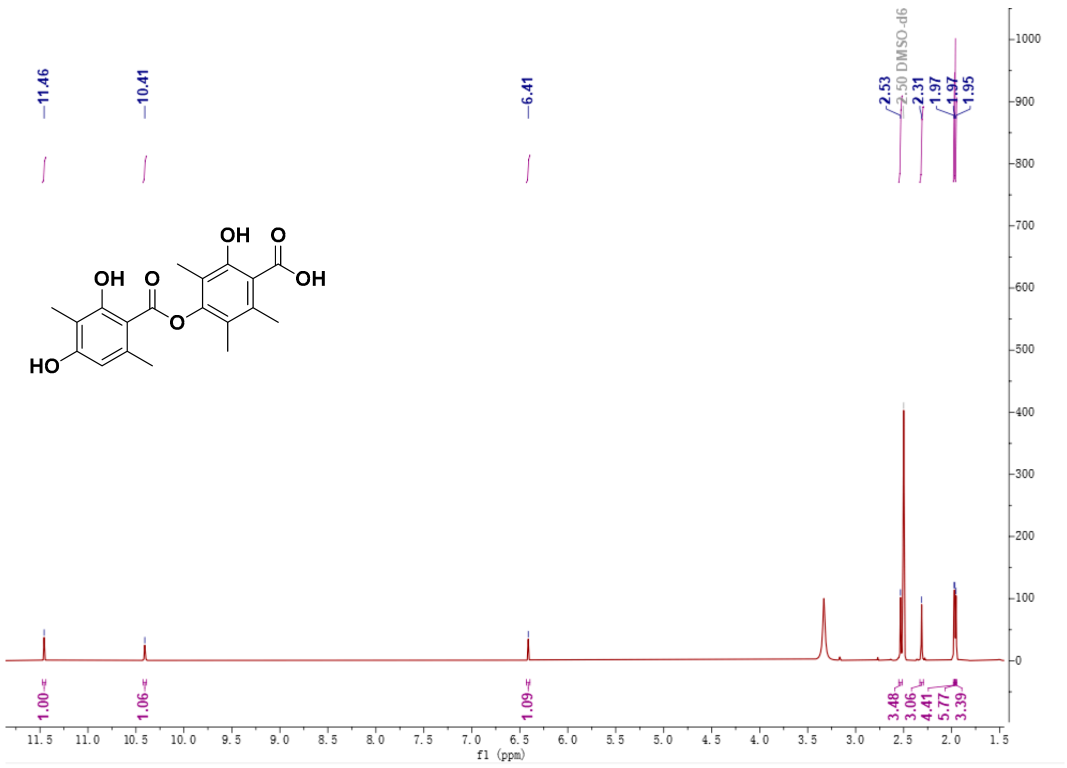

Figure S28 ^{1}H NMR of **5** in DMSO-*d6*

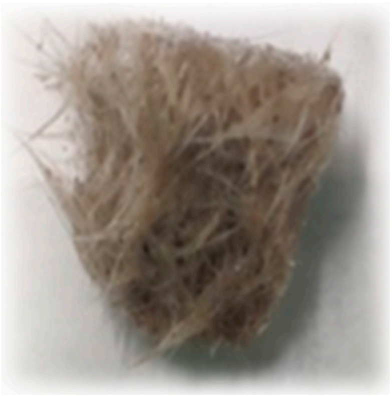

Figure S29. Picture of the isolation source of *Talaromyces* sp. HDN1820200
